# β-Hydroxybutyrate Attenuates Painful Diabetic Neuropathy *via* Restoration of the Aquaporin-4 Polarity in the Spinal Glymphatic System

**DOI:** 10.3389/fnins.2022.926128

**Published:** 2022-07-11

**Authors:** Fei-xiang Wang, Chi-liang Xu, Can Su, Jiang Li, Jing-yan Lin

**Affiliations:** ^1^Department of Anesthesiology, The Affiliated Hospital of North Sichuan Medical College, Nanchong, China; ^2^Department of Medical Imaging, The Affiliated Hospital of North Sichuan Medical College, Nanchong, China

**Keywords:** β-hydroxybutyrate, aquaporin-4, polarity reversal, painful diabetic neuropathy, glymphatic system

## Abstract

Waste removal is essential for maintaining homeostasis and the normal function of the central nervous system (CNS). The glymphatic system based on aquaporin-4 (AQP4) water channels on the endfeet of astrocytes is recently discovered as the excretion pathway for metabolic waste products of CNS. In the CNS, α-syntrophin (SNTA1) directly or indirectly anchors AQP4 in astrocyte membranes facing blood vessels. Studies have indicated that β-hydroxybutyrate (BHB) can raise the expression of SNTA1 and thus restoring AQP4 polarity in mice models with Alzheimer’s disease. The study aims to evaluate the neuroprotective mechanism of BHB in rats with painful diabetic neuropathy (PDN). PDN rats were modeled under a high-fat and high-glucose diet with a low dose of streptozotocin. Magnetic resonance imaging (MRI) was applied to observe the clearance of contrast to indicate the functional variability of the spinal glymphatic system. Mechanical allodynia was assessed by paw withdrawal threshold. The expressions of SNTA1 and AQP4 were tested, and the polarity reversal of AQP4 protein was measured. As demonstrated, PDN rats were manifested with deceased contrast clearance of the spinal glymphatic system, enhanced mechanical allodynia, lower expression of SNTA1, higher expression of AQP4, and reversed polarity of AQP4 protein. An opposite change in the above characteristics was observed in rats being treated with BHB. This is the first study that demonstrated the neuroprotective mechanism of BHB to attenuate PDN *via* restoration of the AQP4 polarity in the spinal glymphatic system and provides a promising therapeutic strategy for PDN.

## Introduction

Painful diabetic neuropathy (PDN) is one of the most common complications of diabetes (Schmader, 2002; Schreiber et al., 2015; Iqbal et al., 2018). It is estimated that approximately 30–50% of patients with diabetic neuropathy develop neuropathic pain, which most commonly takes the form of spontaneous (that is, stimulus-independent) burning pain of the feet or other positive sensory symptoms, such as brush-evoked allodvnia (when a normally non-noxious stimulus evokes pain) and paresthesias (Feldman et al., 2019). Sleeping disorders, fatigue, decreased activity, reduced quality of life, and high medical costs are reported as the negative effects of PDN (Galer et al., 2000; Schmader, 2002; Feldman et al., 2019). The underlying mechanism of PDN is not well understood and the therapeutic effects of current treatments are unsatisfactory, which mainly focused on patients’ symptoms (Javed et al., 2015; Iqbal et al., 2018). Studies have shown that a series of metabolic disorders occurred during the course of diabetic neuropathy (Hussain and Adrian, 2017). It is reported that the production of reactive oxidative species (ROS), activated proinflammatory transcription factors, impaired glutamate (excitatory neurotransmitter) clearance, and hyperexcitability of sensory neurons are involved in the process of PDN (Rivera-Aponte et al., 2015; Feldman et al., 2019; Rendra et al., 2019). These results suggest that abnormal metabolites may be involved in the occurrence and development of pain.

Waste removal is important for maintaining the normal function of the central nervous system (CNS), which is sensitive to the changes in the surrounding environment and lacks authentic lymphatic vessels (Carare et al., 2014; Jessen et al., 2015). The glymphatic system based on aquaporin-4 (AQP4) water channels on the endfeet of astrocytes is recently discovered as the excretion pathway for metabolic waste products of CNS (Plog and Nedergaard, 2018; Davoodi-Bojd et al., 2019; Reeves et al., 2020). The glymphatic system of the spinal cord has been confirmed by many studies (Benveniste et al., 2017; Wei et al., 2017; Liu et al., 2018). It is reported that many diseases are associated with the impaired glymphatic system, such as Alzheimer’s disease, traumatic brain injury, and cognitive deficiency associated with diabetes (Kress et al., 2014; Peng et al., 2016; Jiang et al., 2017).

Aquaporin-4 is the main water channel protein expressed in CNS. AQP4 is densely expressed in the endfeet of astrocytes and is an important factor in CNS water and potassium homeostasis (Vandebroek and Yasui, 2020). In the CNS, α-syntrophin (SNTA1) directly or indirectly anchors AQP4 in astrocyte membranes facing blood vessels, and the absence of SNTA1 can reverse the normal polarity distribution of AQP4 toward blood vessels (polarity reversal; Neely et al., 2001; Mestre et al., 2018). Changes in AQP4 expression and localization have been manifested in several CNS disorders, including traumatic brain injury, Alzheimer’s disease, chronic pain, and cognitive deficiency associated with diabetes (Kress et al., 2014; Peng et al., 2016; Jiang et al., 2017; Lu G. et al., 2020).

β-Hydroxybutyrate (BHB), an inhibitor of class I histone deacetylases (HDACs), is a ketone body metabolite that is mainly derived from the brain, liver, heart, and skeletal muscles in times of caloric restriction, fasting or low-carbohydrate ketogenic diet (Newman and Verdin, 2017; Huang et al., 2018). It has been reported to ameliorate proinflammatory responses in many metabolic disorders, including Parkinson’s disease, Alzheimer’s disease, lipopolysaccharide-induced neuroinflammation and memory impairment, alcoholic hepatitis, and microvascular hyperpermeability in diabetes (Chen Y. et al., 2018; Huang et al., 2018; Shippy et al., 2020; Li et al., 2021). Studies have indicated that BHB can raise the expression of SNTA1 and thus restoring AQP4 polarity in mice models with Alzheimer’s disease (Wang et al., 2019). However, whether BHB can target the restoration of AQP4 polarity to improve PDN remains unknown.

In this study, we established a rat model of PDN and treated it with BHB and applied gadolinium-diethylenetriamine penta-acetic acid (Gd-DTPA), a classic Magnetic resonance imaging (MRI) tracer, to investigate the functional variations of the spinal glymphatic system since we were unable to acquire the more rapid and extensive H217O tracer (Alshuhri et al., 2021; Salman et al., 2021). Mechanical allodynia was assessed by paw withdrawal threshold. Furthermore, the expressions of AQP4 and SNTA1, which determine the polarization of AQP4 in the spinal cord, were assessed. The polarity reversal of AQP4 protein was measured by immunofluorescence. This study lays the foundation for the neuroprotective mechanism of BHB and provides a promising therapeutic strategy for PDN.

## Materials and Methods

### Animal and Treatment

Male adult Sprague-Dawley rats (*n* = 50, bodyweight 160–180 *g*) were purchased and housed one per cage in the laboratory animal center of North Sichuan Medical College with *ad libitum* access to food and water on a 12-h light–dark cycle schedule. All experiments have been approved by the ethic committee of North Sichuan Medical College (permit number: NSMC202184) and were in accordance with the ARRIVE guidelines on the Care and Use of Experimental Animals.

**FIGURE 1:**
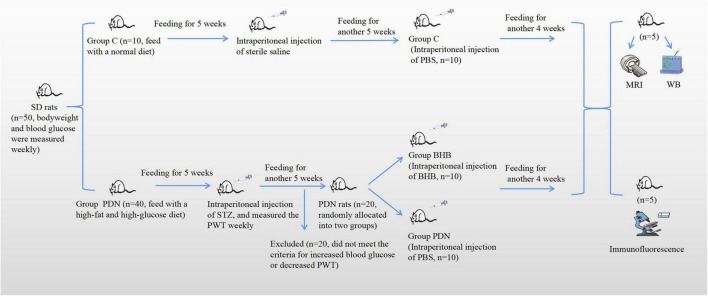
The brief flowchart of the experimental design. Rats were fed for a total of 14 weeks. At the fifth week of feeding, rats in Group PDN were intraperitoneally injected with streptozotocin (STZ) and rats in Group C were intraperitoneally injected with sterile saline. At the 10th week of feeding, there were a total of 20 rats in Group PDN that were considered successfully modeled as PDN rats, and then they were randomly allocated into two groups (Group PDN, *n* = 10; Group BHB, *n* = 10). Afterward, rats in Group BHB were intraperitoneally injected with β-hydroxybutyrate (BHB), whereas rats in Group PDN and Group C were intraperitoneally injected with phosphate-buffered saline (PBS). It should be noted that at the end, 5 rats in each group underwent MRI and WB experiments successively. Finally, MRI, WB, and immunofluorescence were used to detect changes in function, expression level, and expression location. PWT means paw withdrawal threshold.

The rats were randomly allocated into the control group (Group C, *n* = 10, feed with a normal diet) and the PDN modeling group (Group PDN, *n* = 40, feed with a high-fat and high-glucose diet). The modeling process of PDN was in accordance with our previous study (Lin et al., 2018). In brief, after feeding for 5 weeks, diabetes was induced by a single intraperitoneal injection of 25 mg/kg streptozotocin (STZ, Sigma, St. Louis, United States) after an overnight fast. Blood glucose was measured from the tail tip using a one-touch glucometer and matched glucose strips (Johnsons & Johnsons, NJ, United States). Diabetic rats were confirmed with fasting blood glucose (FBG) ≥16.7 mmol/L after 72 h injection. The above modeling process has been confirmed to produce a rat model with diabetes mellitus by multiple studies (Lin et al., 2018; Liu Y. et al., 2019; Huang et al., 2020; Zhang et al., 2020). A brief flowchart of the experimental design is shown in Figure 1.

### Behavioral Tests

Mechanical allodynia was assessed by paw withdrawal threshold (PWT) using the Von Frey filaments kit (US PAT. 5823969 8512259, North Coast, CA, United States) as described previously (Peng et al., 2010; Mei et al., 2011; Lin et al., 2017). The PWT was measured every 7 days after the successful establishment of the diabetic rat model. Briefly, the Von Frey filaments were applied perpendicularly to the hind paw with increasing pressures to induce a positive withdrawal response. The maximum pressure was less than or equal to 26 *g* for avoiding possible injury to the hind paw. Each pressure lasted for 5 s and was repeated four times at different sites of the hind paw, and there was an interval of 15 s between two stimulates. A total of two additional stimulations at that pressure were applied to the hind paw if one positive response was induced. The pressure that could induce three times withdrawal responses in six stimulations was recorded as the PWT. The PWT measurements were taken by the same investigators from 9 to 11 am on experimental days in the same room.

Then, 5 weeks after measurement of FBG, when the animal models were mature and stable, there were a total of twenty rats in the Group PDN manifested increased FBG, decreased PWT and polydipsia, polyuria, and polyphagia, and these rats were considered successfully modeled as PDN rats. Then, the PDN rats were randomly allocated into the PDN group (Group PDN, *n* = 10) and the BHB treatment group (Group BHB, *n* = 10). Finally, the three groups of 10 rats each were used for further research.

### β-Hydroxybutyrate Intraperitoneal Injections

In the present study, PDN rats manifested a significant decrease in PWT from the sixth week. Based on this, we continued to feed for another 4 weeks until the mechanical allodynia stabilized at the 10th week and conducted the BHB intraperitoneal injection. BHB (Yinxing laboratory, Shanghai, China) was dissolved in 0.1 M phosphate-buffered saline (PBS, pH 7.4) to a concentration of 10% w/v as a stock solution. Intraperitoneal injections were performed by the same person at 9 am one time a day for 28 consecutive days on Group BHB, Group PDN, and Group C: (1) Group BHB (291.2 mg/kg BW), (2) Group PDN and Group C PBS (291.2 mg/kg BW; Thaler et al., 2010).

### Magnetic Resonance Imaging Measurement

The MRI measurement was performed using a 3.0T clinical MR system (Discovery MR750, GE, United States) equipped with an eight-channel rat special coil. When the intraperitoneal injections of each group were finished, five rats in each group were randomly selected for MRI measurement. All rats were anesthetized with sevoflurane and maintained using oxygen (2 L/min) with sevoflurane (2.0–2.5%) throughout the experiment (Qi et al., 2018). The spinal subarachnoid space injection was applied whereas rats were placed prone on the operating table and the lower abdomen was raised, with L6, a blunt protrusion in the lower part of the spine, as the localization marker, and tail flutter or sudden lateral sway as the success marker (Zhong-xian et al., 2004; Xu et al., 2006). We finally chose the contrast agent with a concentration of 7.5% Gd-DTPA (75 μl Gd-DTPA was diluted with 925 μl sterile saline) for the MRI experiment, because the contrast agent with this concentration had the best development effect after repeated experiments. A total of 25 μl of diluted Gd-DTPA was injected into the L4/5 subarachnoid space at a constant rate (5 min). After the injection, the needle was left *in situ* for 3 min to prevent reflux, and then, the rat was transferred to the coil for MRI scanning after removing the needle (Lam et al., 2017).

The rats’ heart rate and rectal temperature were continuously monitored throughout the scanning and maintained the heart rate at about 328 ± 10 beats per minute. The rectal temperature was maintained at about 37 ± 0.5°C using a feedback-controlled air heating blower (Rapid Electric, Brewster, NY, United States; Wood et al., 2001). T1-weighted MRI using a fast spin-echo sequence was used for scanning the inflowing into and draining out of the contrast agent to the spinal cord before Gd-DTPA injection and for 15, 30 min, 1, 1.5, 2, 2.5, 3, and 6 h after Gd-DTPA injection. By observing the distribution of contrast agent in the spinal cord at different time points, we could obtain the change in contrast agent over time in different groups of rats, and by measuring the MRI signal intensity (SI), we could make a statistical comparison between groups. Rehydration was vital for life maintenance during anesthesia, so the rats were allowed to wake up and drink freely between measurements. The parameters were as follows: TR: 450.0 ms, TE: 5.4 ms, slice thickness: 2.0 mm, slice space: 1.0 mm: FOV: 6.5 cm × 6.5 cm, matrix: 256 × 256, flip angle: 90, bandwidth: 31.25 kHz, frequency direction: A/P.

The MRI SI of each selected picture was measured at the same region of interest (ROI, area was 0.04 cm^2^). The ROI was determined by repeated experiments for it could basically cover the whole gray matter of the spinal cord, and even if there was some deviation in positioning the ROI, the mean value of its signal would not change by more than 100, which had no impact on the statistical results. Besides, to ensure the consistency of measurement location, the 13th thoracic vertebra of rats was taken as the center, and 3 vertebrae were extended up and down, respectively, as the scanning area (Qian and Hui, 2010). An image that clarified where the ROI was drawn on MRI was included in the Supplementary Material.

For a more accurate measurement of the MRI SI, we used the RadiAnt DICOM Viewer (64 bit; Version 2021.1, Medixant, Poland) and the SI was measured by two people who were not aware of the experimental group, and the average of their results was taken as the SI of the picture. At each time point described above, three adjacent typical lumbar enlargement pictures were selected. The average of the results of the three pictures was recorded as the SI of the rats.

### Western Blot

Expressions of SNTA1 and AQP4 protein in the L5 lumbar cord segments were evaluated by western blot. Then, 2 days after MRI scanning, the five rats that completed the MRI scans in each group were anesthetized with 50 mg/kg of 10% chloral hydrate intraperitoneally and the L5 lumbar cord segments were dissected according to the positions of the spinal nerve roots (Lin et al., 2018). Total protein extracts (30 μg) were loaded onto a 10% sodium dodecyl sulfate-polyacrylamide gel, separated by electrophoresis, and transferred onto a polyvinylidene difluoride membrane (Hybond, Escondido, United States). After blocking with 5% non-fat milk powder for 2 h, the membrane was incubated with rabbit anti-AQP4 antibody (1:2,500, Novus, NBP1-87679, Shanghai, China), mouse anti-SNTA1 antibody (1:1,000, Santa Cruz, sc-166634, TX, United States) or rabbit anti-β-actin antibody (1:100,000, ABclonal, AC026, Wuhan, China) overnight at 4°C and then followed by washing and the incubation of the biotin-conjugated goat anti-rabbit antibody (1:5,000, Affinity, s0001, United States) and goat anti-mouse antibody (1:5,000, Abcam, ab6789, Cambridge, United Kingdom) for 3 h at room temperature. The probed target proteins were then visualized with an enhanced chemiluminescence reagent (Zen-bio, 17046, Chengdu, China) and exposed on an X-ray film. The images were acquired by Shanghai Tianeng GIS chassis control software V2.0. Additionally, for western blot experiments, the specificity of the primary antibodies was demonstrated by the detection of bands in the expected molecular weight.

Due to the reason that some blots in the original saved images were not mentioned in this study, we spliced the blots and the typical ones were chosen to indicate the differences in the expression of proteins. To measure the relative expression of proteins more accurately, we homogenized each control blot and defined its value as 1, then quantified relative to control within each blot, and then put the blots together.

### Tissue Blocks and Section

The rest of the five rats in each group were selected for immunofluorescence. The rats were anesthetized with 50 mg/kg of 10% chloral hydrate intraperitoneally and immobilized by transcardial perfusion with 200 ml of sterile normal saline and 200 ml of 4% paraformaldehyde in 0.1 M phosphate-buffered saline (PBS, PH 7.4). Afterward, the L5 lumbar cord segments were dissected according to the positions of the spinal nerve roots of the cauda equina and were postfixed in 4% paraformaldehyde for 2 days (Lin et al., 2018). After being embedded in paraffin (Histowax; Leica Instrumental Ltd, Shanghai, China), tissue blocks were sectioned in the direction perpendicular to the long axis of the spinal cord with a thickness of 4 μm and three sections were sampled from fifteen serial sections according to a systematic (equal spaced) manner.

### Immunofluorescence

After standard deparaffinization, rehydration, and boiling repair antigen, sections were blocked for 60 min in 5% normal goat serum. Then, sections were incubated overnight at 4°C with AQP4 antibody (1:150, Novus, NBP1-87679, Shanghai, China) and PECAM-1/CD31 antibody (1:50, Santa Cruz, sc-376764, Texas, United States). The following day, Alexa Fluor 647 or Alexa Fluor 488 was applied prior to coverslipping with anti-fluorescence quenching sealing solution with DAPI (Beyotime, p0131, Shanghai, China). Finally, the section was mounted and observed under an Olympus FV1200 confocal laser scanning microscope with a 40× objective lens. The parameters were as follows: DAPI: HV 477, Gain 2, Offset 22; Alexa Fluor 488: HV 555, Gain 1, Offset 59; Alexa Fluor 647: HV 466, Gain 1, Offset 38. The typical images were selected to indicate the polarity reversal of AQP4 protein. For an unbiased representation of the images, all laser power parameters, pinhole size, and image detection were kept constants for all samples.

### Quantification of Aquaporin-4 Polarization

A total of three randomly selected sections/animals in each group were analyzed by investigators blinded to treatment conditions using ImageJ version 1.53e bundled with Java 1.8.0_172 software (National Institutes of Health). In brief, we first split the channels of the image and chose the channel of AQP4. To avoid the influence of non-specific staining motor neurons on measurement, the perivascular region was defined as being CD31-positive, having a complete cross-section of blood vessels and no surrounding dendrites. The dendrites were defined as weakly CD31-positive areas outside the perivascular region that looked like branches and were irregularly shaped and disordered. Afterward, the corresponding perivascular region was identified on the AQP4 channel, then, the threshold of the AQP4 channel was automatically assigned, and the median immunofluorescence intensity of the perivascular region was measured. Finally, we used the threshold analysis to measure the percentage of the region exhibiting AQP4 immunofluorescence lower than perivascular AQP4 immunofluorescence of the whole image, and the proportion was expressed as AQP4 polarization (Harrison et al., 2020). The ventral horn, the dorsal horn, and the central canal on each selected section were measured. The average of the five results on each section was recorded as the AQP4 polarization of the section. The mean of the three randomly selected sections was recorded as the AQP4 polarization of the animal.

### Statistical Analysis

Statistical analyses were performed using the SPSS software version 23 (IBM Corporation, Armonk, NY). A value of 0.05 was used to evaluate the significance of all tests. All values were shown as mean ± SEM. The one-way analysis of variance (ANOVA) was used to compare means between groups. The repeated-measures ANOVA was used to compare the PWT and MRI signal intensity. *Post hoc* analyses were conducted using the Bonferroni correction method.

## Results

### Brief Flowchart of Experimental Design

In this study, all rats were fed for a total of 14 weeks. Rats in Group C were fed with a normal diet and rats in Group PDN were fed with a high-fat and high-glucose diet. At the fifth week of feeding, rats in Group PDN were intraperitoneally injected with STZ, and rats in Group C were intraperitoneally injected with sterile saline. At the 10th week of feeding, there were a total of 20 rats in Group PDN that were considered successfully modeled as PDN rats, and then, they were randomly allocated into two groups (Group PDN, *n* = 10; Group BHB, *n* = 10). Afterward, rats in Group BHB were intraperitoneally injected with BHB, whereas rats in Group PDN and Group C were intraperitoneally injected with PBS. It should be noted that at the end, 5 rats in each group underwent MRI and WB experiments successively. Finally, MRI was used to detect the changes in contrast clearance of the spinal glymphatic system, western blot was used to measure the expression of proteins of L5 lumbar cord segments, and immunofluorescence was used to demonstrate changes in AQP4 localization (Figure 1).

### Bodyweight and Blood Glucose in Each Group of Rats

**FIGURE 2:**
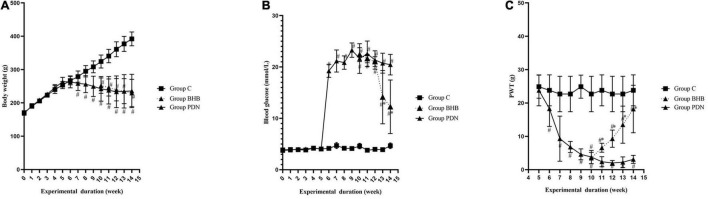
Trend of bodyweight, blood glucose, PWT in each group. **(A)** The trend of bodyweight in Group C, Group PDN, and Group BHB. **(B)** The trend of blood glucose in Group C, Group PDN, and Group BHB. **(C)** The trend of PWT in Group C, Group PDN, and Group BHB. At the fifth week of feeding, rats in Group PDN were intraperitoneally injected with STZ, and rats in Group C were intraperitoneally injected with sterile saline. Rats in Group BHB were intraperitoneally injected with STZ at the fifth week of feeding and BHB at the 10th week of feeding. The one-way analysis of variance (ANOVA) was used to compare means between groups. The repeated-measures ANOVA was used to compare the PWT. *Post hoc* analyses were conducted using the Bonferroni correction method. Values were presented as mean ± SEM. *p < 0.05 compared with values in Group PDN. ^#^*p* < 0.05 compared with values in Group C.

As shown in Figure 2A, for the entire duration of the experiment, the bodyweight of rats in Group PDN manifested a trend of rising first and then falling while in Group C manifested a trend of rising. Rats in Group PDN started to increase water and food consumption and urine output 3 days after injection of STZ, as well as lose body weight, which are typical symptoms of diabetes mellitus (polydipsia, polyphagia, and polyuria). From the seventh week of feeding, the bodyweight of rats in Group C and group PDN was statistically significant compared with each other (*p* < 0.05). The bodyweight of rats in Group PDN at the seventh week of feeding and rats in Group BHB at the 10th week of feeding started to decrease significantly when compared with Group C (*p* < 0.05). From the sixth week of feeding until the end of the experiment, the blood glucose of rats in Group PDN increased significantly when compared with Group C (*p* < 0.05). It is worth mentioning that the blood glucose of rats in Group BHB decreased significantly after 2 weeks of BHB intraperitoneal injection when compared to Group PDN (*p* < 0.05). Decreased bodyweight and increased blood glucose indicated that the modeling process of diabetic rats was successfully completed (Figure 2B).

### Paw Withdrawal Threshold in Each Group of Rats

Compared to Group C, the PWT decreased significantly in Group PDN 7 days after FBG was tested and lasted until the end of the experiment (*p* < 0.05). The PWT increased significantly in Group BHB after 1 week of BHB intraperitoneal injection compared with values in Group PDN (*p* < 0.05), which indicated that BHB can attenuate mechanical allodynia (Figure 2C).

### Magnetic Resonance Imaging

#### Dynamic Observation of Gadolinium-Diethylenetriamine Penta-Acetic Acid Inflowing Into and Draining Out of the Spinal Cord

**FIGURE 3:**
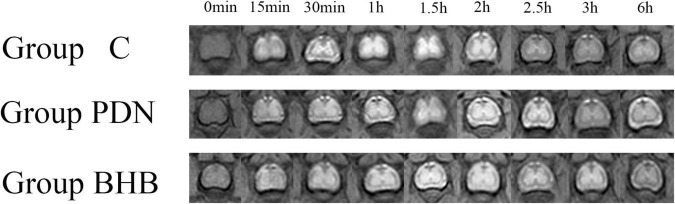
The MRI image with time in lumbar enlargement of the spinal cord of rats in Group C, Group PDN, and Group BHB before and after 25 μl diluted gadolinium-diethylenetriamine penta-acetic acid (Gd-DTPA, 75 μl Gd-DTPA was diluted with 925 μl sterile saline) injection (0 min–6 h) through the lumbar 4/5 intervertebral space. Refer to Figure 1 for the meaning of the grouping abbreviation.

The SI of the lumbar enlargement of the spinal cord changed dynamically with time. Before Gd-DTPA injection, there was no contrast agent in the lumbar enlargement of rats in each group. After Gd-DTPA injection, the contrast agent was started to discontinuously distribute on the surface of the pia mater in the lumbar enlargement of rats in each group, and the contrast agent was concentrated in gray matter, whereas no contrast agent was observed in white matter. Over time, the contrast agent began to enter the spinal gray matter along the anterior lateral sulcus or the posterior lateral sulcus and showed the brightest “butterfly shape” when its net intake was at its maximum and then began to darken (Figure 3).

#### Measurement Results of Signal Intensity

To make the data more comparable, we defined the mean of SI before Gd-DTPA injection in Group C as 100. As shown in Table 1, between the three groups, there was no statistical difference in SI values before Gd-DTPA injection, the peak of SI (PEAK SI), and the SI in the lumbar enlargement 6 h after finishing Gd-DTPA injection (SIX SI). The variation in the SI per hour after SI reached its peak (SIPH) decreased significantly in the Group PDN (6.99 ± 0.61) when compared to Group C (10.26 ± 2.44) whereas SIPH increased significantly in the Group BHB (10.00 ± 0.74) when compared to Group PDN (*p* < 0.05). The time to reach PEAK SI (PEAK TIME) in each group was not statistically different compared with each other.

**TABLE 1:** MRI signal intensity (SI) obtained from the lumbar enlargement.

	Group C (*n* = 5)	Group PDN (*n* = 5)	Group BHB (*n* = 5)	*p*-value
Pre-injection	100.00 ± 14.99	118.19 ± 25.45	116.97 ± 19.76	0.327
PEAK SI	186.59 ± 32.88	198.71 ± 30.74	201.13 ± 33.92	0.772
SIX SI	142.68 ± 37.37	166.81 ± 32.20	162.09 ± 33.42	0.488
SIPH	10.26 ± 2.44	6.99 ± 0.61*	10.00 ± 0.74^#^	0.009
PEAK TIME	1.90 ± 0.82	1.90 ± 0.65	2.00 ± 0.50	0.964

*Group C: Rats intraperitoneally injected with sterile saline and phosphate-buffered saline. Group PDN: Rats intraperitoneally injected with streptozotocin and phosphate-buffered saline. Group BHB: Rats intraperitoneally injected with streptozotocin and β-Hydroxybutyrate. Pre-injection: the SI before Gadolinium-diethylenetriamine Penta-acetic acid (Gd-DTPA) injection. PEAK SI: the peak of SI. SIX SI: the SI 6 h after finishing the Gd-DTPA injection. SIPH: the variation of the SI per hour after SI reached its peak. PEAK TIME: the time to reach PEAK SI.*

*p < 0.05 compared with values in Group C.

*^#^p < 0.05 compared with values in Group PDN.*

#### Expressions of Aquaporin-4 and SNTA1 Protein

**FIGURE 4:**
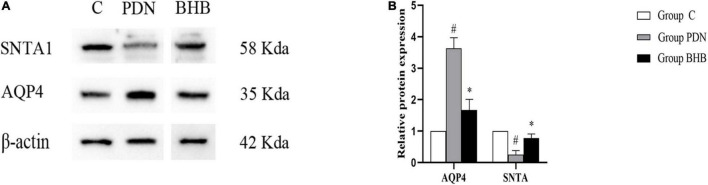
Western blot results. **(A)** Western blot analysis of SNTA1 and AQP4 protein expression in Group C, Group PDN, and Group BHB. **(B)** Relative protein expression of SNTA1 and AQP4. Due to the reason that some blots in the original saved images were not mentioned in the present study, we spliced the blots and the typical ones were chosen to indicate the differences in the expression of proteins. Refer to Figure 1 for the meaning of the grouping abbreviation. Values were presented as mean ± SEM. *p < 0.05 compared with values in Group PDN. ^#^*p* < 0.05 compared with values in Group C. SNTA1 means α-syntrophin, and AQP4 means aquaporin-4 protein.

Compared with Group C, the expression of AQP4 protein was higher (*p* < 0.05) and the expression of SNTA1 protein was lower (*p* < 0.05) in Group PDN. Compared with the Group PDN, the expression of AQP4 protein was lower (*p* < 0.05) and the expression of SNTA1 protein was higher (*p* < 0.05) in Group BHB (Figure 4 and Table 2).

**TABLE 2:** Immunofluorescence quantification and Western blot results obtained from the spinal cord.

	Group C (*n* = 5)	Group PDN (*n* = 5)	Group BHB (*n* = 5)	*p*-value
Relative AQP4	1.00 ± 0.00	3.63 ± 0.33*	1.67 ± 0.34^#^*	<0.001
Relative SNTA1	1.00 ± 0.00	0.25 ± 0.13*	0.72 ± 0.13^#^*	<0.001
AQP4 polarization (%)	68.07 ± 6.95	33.60 ± 5.54*	63.33 ± 13.24^#^	<0.001

*See Table 1 for the meaning of the grouping abbreviation. AQP4: aquaporin-4; SNTA1: α-syntrophin.*

*P < 0.05 compared with values in Group C.

*^#^P < 0.05 compared with values in Group PDN.*

#### Polarity Localization of Aquaporin-4 Protein

**FIGURE 5:**
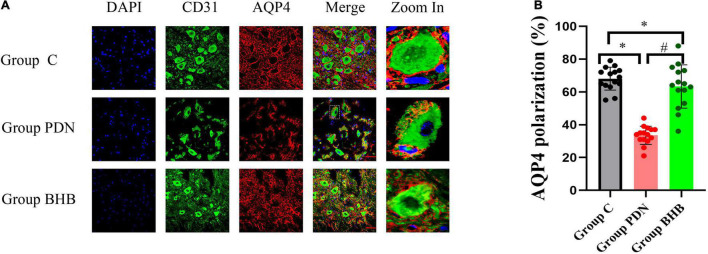
Immunofluorescence results. **(A)** Immunofluorescence staining of sections, using anti-CD31 and anti-AQP4 antibodies, was obtained from the L5 segment of the spinal cord of rats in Group C, Group PDN, and Group BHB. Refer to Figure 1 for the meaning of the grouping abbreviation. AQP4-positive cells were identified with red fluorescence, CD31-positive cells were identified with green fluorescence, and the nuclei were counterstained with DAPI (blue). A typical part of AQP4 protein polarity reversal was zoomed in and shown in detail. Scale bar = 50 μm. **(B)** Quantification of AQP4 polarization similarly demonstrated the polarity reversal of PDN rats and BHB can restore the reversed polarization. *p < 0.05 compared with values in Group C. ^#^*p* < 0.05 compared with values in Group PDN.

In this study, we used the additional vascular endothelial cell markers CD31 to determine the polarity localization of AQP4 protein (Stokum et al., 2015). As shown in Figure 5 and Table 2, the reduced perivascular localization of AQP4 protein of rats in Group PDN was concordant with a reduction in “polarization” of this water channel to blood vessels [AQP4 “polarization” of rats in Group C, 68.07 (±6.95%) versus 33.60% (±5.54%) of rats in Group PDN, *p* < 0.05], which indicated the polarity reversal of AQP4 protein in Group PDN. The restored perivascular localization of AQP4 protein of rats in Group BHB was concordant with an increase of “polarization” of this water channel to blood vessels [AQP4 “polarization” of rats in Group BHB, 63.33 (±13.24%) versus 33.60% (±5.54%) of rats in Group PDN, *p* < 0.05]. The above results indicated the polarity reversal of AQP4 protein in Group PDN and the restored polarization of AQP4 protein in Group BHB.

## Discussion

This is the first study that manifested the neuroprotective mechanism of BHB to attenuate PDN *via* restoration of the AQP4 polarity in the spinal glymphatic system. As demonstrated, the manifestation of rats with PDN includes deceased contrast clearance of the spinal glymphatic system, enhanced mechanical allodynia, lower expression of SNTA1, and higher expression of AQP4 protein. The polarity of AQP4 protein reversed significantly in PDN rats. After being treated with BHB, PDN rats were manifested with enhanced contrast clearance of the spinal glymphatic system, attenuated mechanical allodynia, higher expression of SNTA1, lower expression of AQP4 protein, and restored polarity of AQP4 protein. All the above characteristics indicated the neuroprotective function of BHB and provided a promising therapeutic strategy for PDN.

The glymphatic system based on AQP4 water channels on the endfeet of astrocytes is recently discovered as the excretion pathway for metabolic waste products of CNS (Plog and Nedergaard, 2018; Davoodi-Bojd et al., 2019; Reeves et al., 2020). The glymphatic system of the spinal cord has been confirmed by many studies (Benveniste et al., 2017; Wei et al., 2017; Liu et al., 2018). It is reported that many diseases are associated with the impaired glymphatic system, such as Alzheimer’s disease, traumatic brain injury, and cognitive deficiency associated with diabetes (Kress et al., 2014; Peng et al., 2016; Jiang et al., 2017). MRI is widely used to study the functional changes in the glymphatic system because of its high sensitivity and non-invasive evaluation of the fluid exchange of cerebrospinal fluid (CSF) with interstitial fluid (Gakuba et al., 2018; Jiang, 2019; Zhang et al., 2019). In this study, we injected Gd-DTPA into the lumbar enlargement of living PDN rats and mainly observed the clearance of contrast agent to indicate the functional changes in the spinal glymphatic system. Using MRI evaluation, we found that the clearance of contrast agent in the spinal glymphatic system of living PDN rats was deceased, which indicated that the activity of the spinal glymphatic system in PDN rats was impaired. We also noticed that the clearance of contrast agent was enhanced in living PDN rats after being treated with BHB, which indicated that BHB enhanced the clearance of contrast agent in the spinal glymphatic system of PDN rats.

This is the first study using MRI scanning to determine the neuroprotective function of BHB on the spinal glymphatic system during the process of PDN. The SI of the lumbar enlargement of the spinal cord changed dynamically with time. For the entire duration of the experiment, there was no contrast agent signal in white matter in each group. After Gd-DTPA injection, the contrast agent began to distribute discontinuously on the surface of the pia mater and then enter the spinal gray matter along the anterior lateral sulcus or the posterior lateral sulcus. The inflowing and removal of the contrast agent were both present in the whole process and fitted well with previous studies (Wei et al., 2017). These results suggested that the contrast agent may enter the spinal cord through the perivascular space of the radicle arteries and the perforating arteries of the pia mater, and due to the limited resolution of 3.0T MRI and the small spinal cord of rats, we did not find clear evidence to prove that process, and we hope to figure it out in the future study.

The inflowing of contrast agent into the spinal parenchyma was a time-dependent process. In this study, there was no statistical difference in SI values before Gd-DTPA injection between the three groups, and it looked like that the SI values were higher in Group PDN and Group BHB than in Group C. A previous study has confirmed the existence of inflammation in the spinal cord of PDN rats (Liu S. et al., 2019). Considering the small sample size of rats in this study, we believe that there will be a statistical difference in SI values before Gd-DTPA injection between the three groups when the sample size is expanded. Of course, this needs to be confirmed by more studies in the future. The PEAK SI and SIX SI in each group had the same characteristics as the SI values before Gd-DTPA injection, that was, inter-group comparisons were not statistically significant, but the values in Group PDN and Group BHB appeared to be higher than those in Group C. We also noticed that the PEAK TIME in each group was not statistically different compared with each other. The SIPH decreased significantly in Group PDN when compared to Group C whereas the SIPH increased significantly in Group BHB when compared to Group PDN. Previous studies have indicated that the speed of CSF bulk in the para-vasculature network of the spinal cord of PDN rats was lower and the perivascular space in diabetes is enlarged (Jiang et al., 2017; Davoodi-Bojd et al., 2019). Aggregation of glycation end products and inflammation may also increase the perivascular space and lead to stagnant glymphatic transport, so as to result in the deceased clearance of contrast agent in the spinal cord of living PDN rats (Kress et al., 2014; Peng et al., 2016). In conclusion, the contrast agent injected into the spinal cord of the three groups of rats showed a similar trend over time, and we found that the clearance rate of the spinal glymphatic system in PDN rats decreased significantly, and BHB reversed the decrease.

Aquaporin-4 belongs to the family of channels that is selectively permeable to water, and it is the most abundant water channel in the brain, spinal cord, and optic nerve and controls brain water homeostasis (Mader and Brimberg, 2019). It plays an important role in the solute exchange and waste removal in the brain due to its highly selective expression in the blood–brain barrier (BBB) and blood–CSF barrier (Benveniste et al., 2017; Mader and Brimberg, 2019). Many neurological conditions are now found to be associated with an alteration in AQP4 expression or localization, such as CNS inflammation, glymphatic fluid clearance, synaptic plasticity and memory formation, cognitive deficits, regulation of extracellular space (ECS) volume and potassium homeostasis, chronic pain, and neuropathic pain (Nesic et al., 2005; Nagelhus and Ottersen, 2013; Jiang et al., 2017; Hubbard et al., 2018; Lu G. et al., 2020; Lu H. et al., 2020).

In the CNS, SNTA1 directly or indirectly anchors AQP4 in astrocyte membranes facing blood vessels (Neely et al., 2001; Mestre et al., 2018). The absence of SNTA1 diminishes perivascular AQP4 expression and reverses the normal concentration differences between perivascular membranes and other regions (known as AQP4 polarity reversal). BHB, an inhibitor of HDACs, is a ketone body metabolite that is mainly derived from the brain, liver, heart, and skeletal muscles in times of caloric restriction, fasting or low-carbohydrate ketogenic diet (Newman and Verdin, 2017; Huang et al., 2018). Ketosis produced through a ketogenic diet may be an appropriate treatment strategy for persistent pain and dysfunction within the nervous system involving changes in both cortical structure and function (Field et al., 2021). Previous studies have demonstrated the anti-inflammatory and neuroprotective function of BHB in many metabolic disorders, including lipopolysaccharide-induced neuroinflammation and memory impairment, Parkinson’s disease, Alzheimer’s disease, alcoholic hepatitis, and microvascular hyperpermeability in diabetes (Chen Y. et al., 2018; Huang et al., 2018; Shippy et al., 2020; Li et al., 2021). There are also studies indicating that BHB promotes functional recovery and relieves pain hypersensitivity in mice models of spinal cord injury, possibly through inhibition of histone deacetylation and NLRP3 inflammasome activation and preservation of mitochondrial function (Qian et al., 2017; Boccella et al., 2019). In conclusion, in addition to acting as a substrate for energy metabolism, BHB can also participate in the regulation of a series of diseases by directly or indirectly acting as a signaling molecule.

It looked like that there were some differences in the architecture, such as the blood vessels between groups in Figure 5, and we thought that the disrupted binding of CD31 due to vascular endothelial dysfunction and atherosclerosis in the Group PDN might be the cause. In addition, the non-specific staining motor neurons, which acted like dendrites extending off of the blood vessels in Figure 5, often bind indiscriminately to antibodies and may be confused with CD31 in this study. To eliminate these disturbances, we excluded CD31-positive areas that looked incomplete and had dendrites from our measurements when we located the perivascular regions and CD31 was not involved in the measurement of the average fluorescence intensity of AQP4 in the perivascular area. Therefore, the quantification of AQP4 polarization in this study was reliable and believable. After the three channels were merged, it was obvious that in the PDN group, red AQP4 was not closely distributed around the cross-section of blood vessels, but discontinuous, and there was even no AQP4 distribution in some perivascular regions, indicating that the polarity of AQP4 had changed significantly in the Group PDN and BHB can restore the polarity reversal of the AQP4 protein in PDN rats.

The lower expression of SNTA1 protein was matched with AQP4 polarity reversal in Group PDN, since SNTA1 was crucial for the normal polarization of AQP4. The expression of AQP4 protein in Group PDN was higher and combined with the results of Group C, we draw a conclusion that the normal distribution of AQP4 is more important for water homeostasis than the expression level. When the distribution of AQP4 changes, that is, when the polarity is reversed, the body compensates for water homeostasis by increasing the expression of AQP4. A previous study indicated that Sin3-HDAC complexes can regulate the gene expression of SNTA1 (Van Oevelen et al., 2010). The study of Wang et al. has confirmed that BHB can antagonize the decrease of SNTA1 expression by inhibiting histone deacetylase 1 (HDAC1) expression and thus restore AQP4 polarity in mice with Alzheimer’s disease, and it was concordant with the results of Wang et al. (2019) and Li et al. (2020). Besides, in the *in vitro* experiment of astrocytes, Wang et al. have found that silencing the HDAC1 gene could upregulate the mRNA and protein expression of SNTA1 and it fitted well with our results (Wang et al., 2019). In conclusion, we speculate that BHB may inhibit HDAC1 and increase the expression of SNTA1, decrease the expression of AQP4, and restore the normal polarity of AQP4, thus playing a therapeutic role in PDN. In addition, valproic acid, another HDAC inhibitor, has been identified to have anti-inflammatory, antioxidant, neuroprotective, anti-fibrosis, and other effects and has a good therapeutic effect in cerebral ischemia, epilepsy, spinal cord injury, and other diseases (Khan et al., 2016; Chen S. et al., 2018; Silva et al., 2018). Whether valproic acid can also produce a therapeutic effect on PDN by inhibiting HDAC can be used as new evidence to support the role of HDAC in PDN since the inhibitory effect of BHB on HDACs is non-specific, and we hope to conduct this experiment in the future. Anyway, our results indicated that BHB can antagonize the decrease of SNTA1 expression, increase the expression of AQP4, and restore its polarity in PDN rats.

We have noticed that the blood glucose of rats in Group BHB decreased significantly when compared to values in Group PDN, which may be related to BHB playing a key role in sparing glucose utilization (Laffel, 1999). Additionally, it should be noted that the AQP4 expression and its polarity reversal can be two different concepts and can be discordant. For example, previous studies have shown an increase in AQP4 membrane localization in primary human astrocytes which is not accompanied by a change in AQP4 protein expression levels (Salman et al., 2017). This mislocalization can be a potential therapeutic target (Ciappelloni et al., 2019). Studies have demonstrated that AQPs were involved in human physiology and pathophysiology, such as fluid homeostasis and secretion, signal transduction and sensory functions, defense and metabolism, and motility and cancer (Wagner et al., 2022). Recently, the research and development of drugs targeting AQPs to treat diverse conditions have become a hot topic (Verkman et al., 2014; Abir-Awan et al., 2019; Markou et al., 2022). Recent breakthroughs indicate that inhibition of hydrogen peroxide permeation through AQP1 provides a new approach to treat hypertrophic cardiomyopathies, and inhibition of AQP4 localization with the licensed drug trifluoperazine provides compelling evidence for a new approach to treating CNS edema (Salman et al., 2022).

In this study, the injection of the contrast agent into the subarachnoid space may affect the CSF flow, and at the same time, the increased MRI signal-to-noise ratio may help to better detect the functional changes in the spinal glymphatic system and make the quantitative analysis more accurate (Ding et al., 2018). Previous studies have demonstrated that continuous anesthesia with sevoflurane increases AQP4 expression (Gao et al., 2019). In this study, sevoflurane anesthesia is performed intermittently and the rats were allowed to wake up and drink freely between measurements. Most importantly, the duration of anesthesia in different rats was basically the same, and we believe that the influence of anesthesia with sevoflurane on AQP4 expression can be counteracted. Of course, the effect of sevoflurane anesthesia on AQP4 expression in PDN rats needs further study. Previous studies have indicated that there is no correlation between CSF sodium concentration and saline intake, and drinking water does not affect brain total water content (Frankmann et al., 1987; Meyers et al., 2016). In other words, drinking water does not affect the total amount of CSF, so the influence of drinking water on the spinal glymphatic system can be ignored. MRI scanning was not performed for a longer time due to the limitation of using time of the equipment. So, there was no further comparison of the complete clearance time to contrast agent between the three groups, and it also needs further exploration in the future studies.

In summary, this study successfully used low-dose STZ intraperitoneal injection combined with a high-fat and high-glucose diet to establish PDN rats with pathophysiological similarity to human type 2 diabetes mellitus. As demonstrated, the manifestation of rats with PDN includes deceased contrast clearance in the spinal glymphatic system, enhanced mechanical allodynia, lower expression of SNTA1, and higher expression of AQP4 protein. The polarity of AQP4 protein reversed significantly in PDN rats. After being treated with BHB, PDN rats were manifested with enhanced contrast clearance in the spinal glymphatic system, attenuated mechanical allodynia, higher expression of SNTA1, lower expression of AQP4 protein, and restored polarity of AQP4 protein. This is the first study that manifested the neuroprotective mechanism of BHB to attenuate PDN *via* restoration of the AQP4 polarity in the spinal glymphatic system and provides a promising therapeutic strategy for PDN.

## Data Availability Statement

The raw data supporting the conclusions of this article will be made available by the authors, without undue reservation.

## Ethics Statement

The animal study was reviewed and approved by the Ethics Committee of North Sichuan Medical College.

## Author Contributions

J-YL: conceptualization and methodology. F-XW: data curation, formal analysis, and writing—original draft. C-LX: validation and visualization. CS and JL: investigation. All authors read and approved the final manuscript.

## Conflict of Interest

The authors declare that the research was conducted in the absence of any commercial or financial relationships that could be construed as a potential conflict of interest.

## Publisher’s Note

All claims expressed in this article are solely those of the authors and do not necessarily represent those of their affiliated organizations, or those of the publisher, the editors and the reviewers. Any product that may be evaluated in this article, or claim that may be made by its manufacturer, is not guaranteed or endorsed by the publisher.

## Abbreviations

AQP4: aquaporin-4 protein
PDN: painful diabetic neuropathy
ROS: reactive oxidative species
SNTA1: α-syntrophin
BHB: β-hydroxybutyrate
CNS: central nervous system
HDACs: class I histone deacetylases
HDAC1: histone deacetylase 1
STZ: streptozotocin
FBG: fasting blood glucose
PWT: paw withdrawal threshold
Gd-DTPA: gadolinium-diethylenetriamine penta-acetic acid
T1WI: T1-weighted MRI
FSE: fast spin echo
SI: signal intensity
PEAK SI: the peak of SI
SIX SI: the SI in the lumbar enlargement 6 h after finishing Gd-DTPA injection
SIPH: the variation in the SI per hour after SI reached its peak
PEAK TIME: the time to reach PEAK SI
PBS: phosphate-buffered Saline
BBB: blood–brain barrier
ANOVA: one-way analysis of variance
CSF: cerebrospinal fluid
ECS: extracellular space
ISF: interstitial fluid.
