# Supplementary figures and images for: β-Hydroxybutyrate Attenuates Painful Diabetic Neuropathy via Restoration of the Aquaporin-4 Polarity in the Spinal Glymphatic System
Source: Front Neurosci. 2022 Jul 11;16:926128. doi: 10.3389/fnins.2022.926128 (PMC9309893; doi:10.3389/fnins.2022.926128)

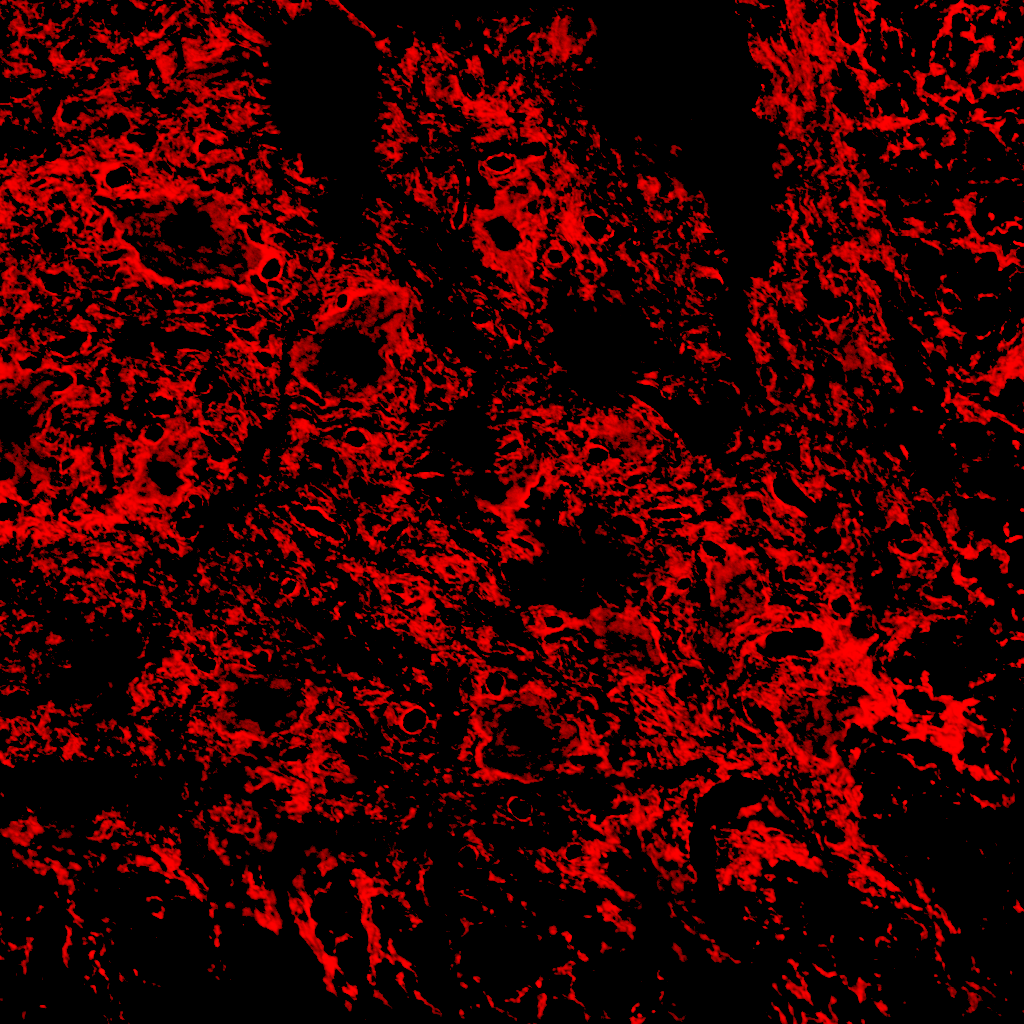

Supplement: Supplementary file 1 [file Data_Sheet_1.zip › Supplementary materials/Immunofluorescence images/Group BHB/AQP4.tif]

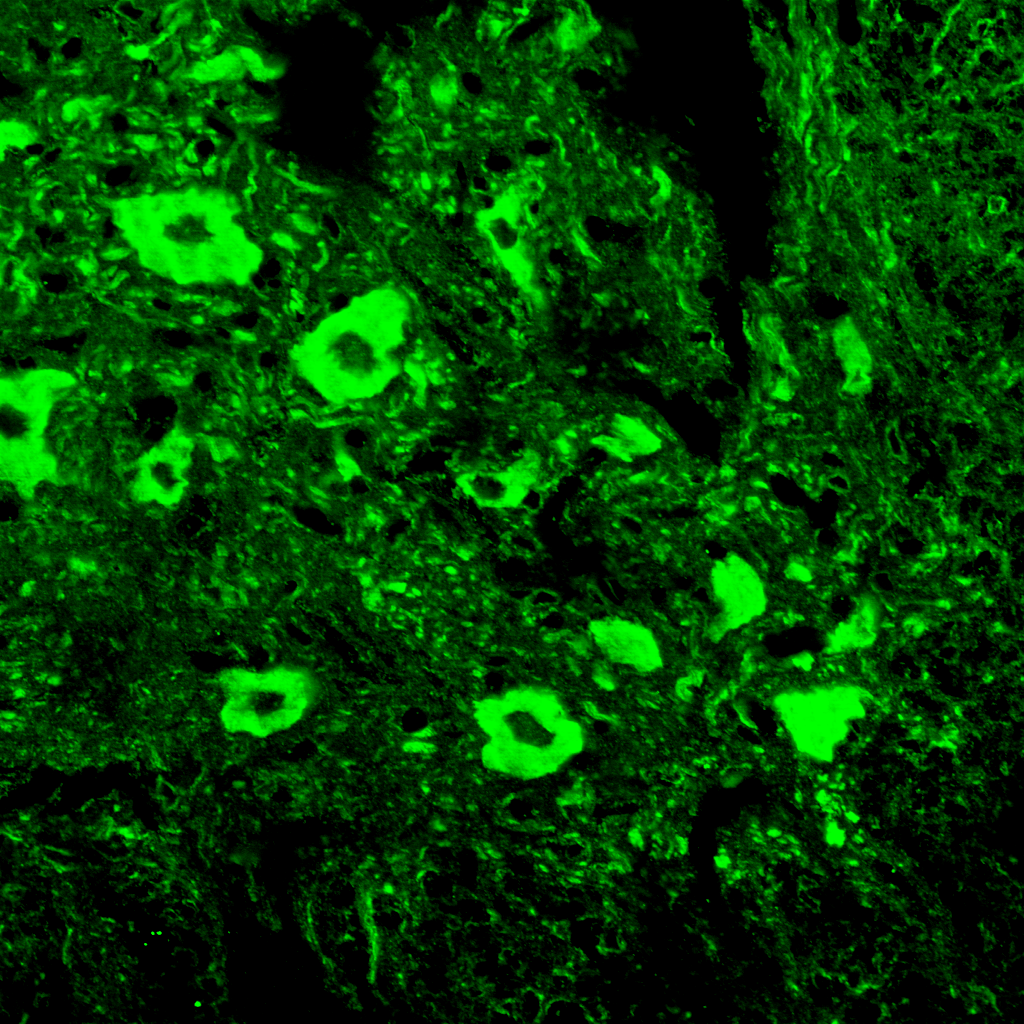

Supplement: Supplementary file 1 [file Data_Sheet_1.zip › Supplementary materials/Immunofluorescence images/Group BHB/CD31.tif]

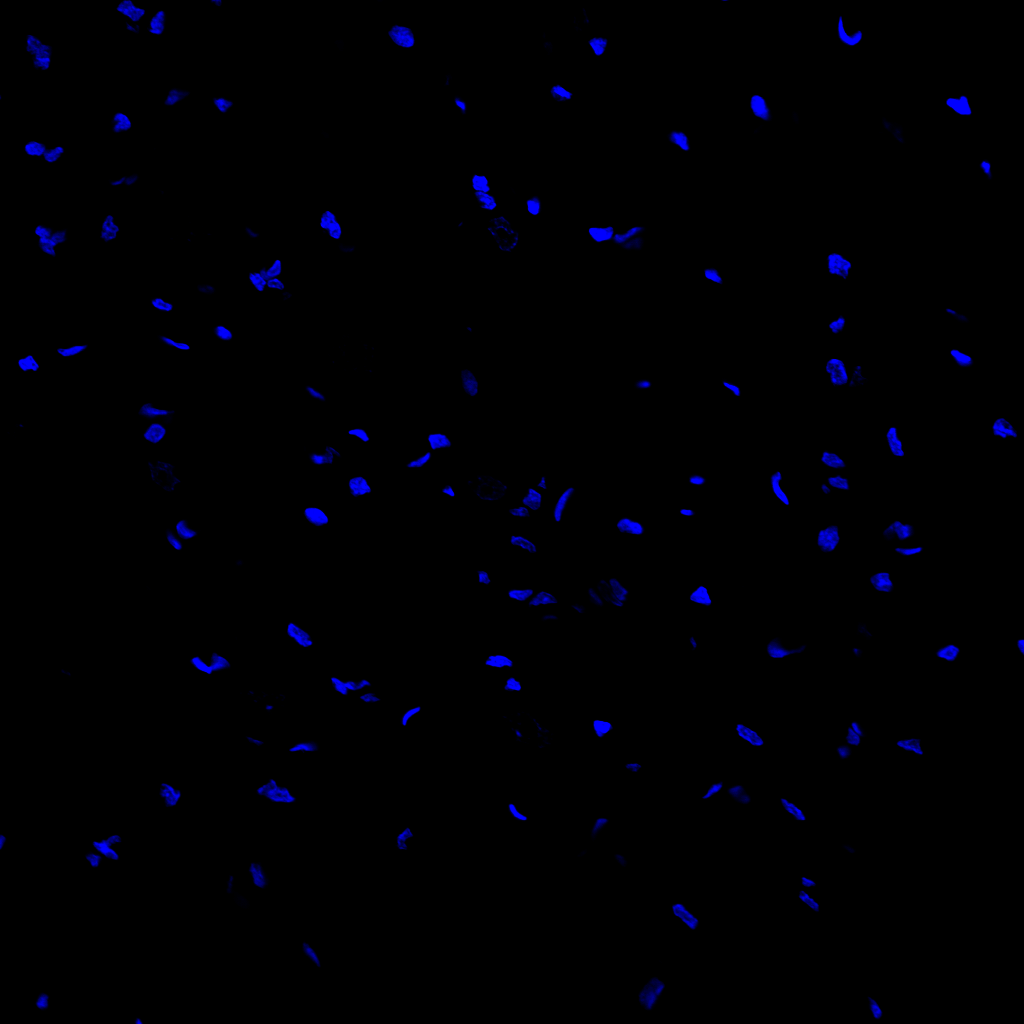

Supplement: Supplementary file 1 [file Data_Sheet_1.zip › Supplementary materials/Immunofluorescence images/Group BHB/DAPI.tif]

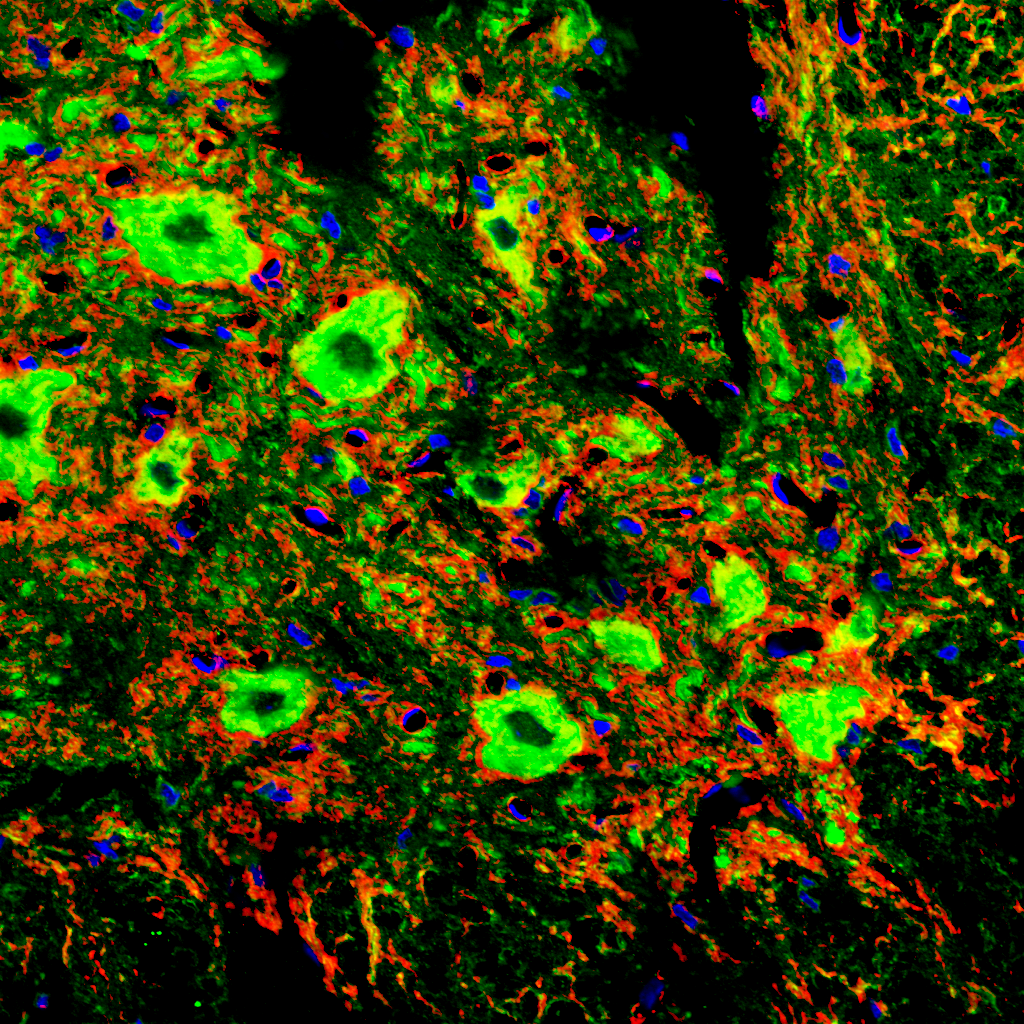

Supplement: Supplementary file 1 [file Data_Sheet_1.zip › Supplementary materials/Immunofluorescence images/Group BHB/Merge.tif]

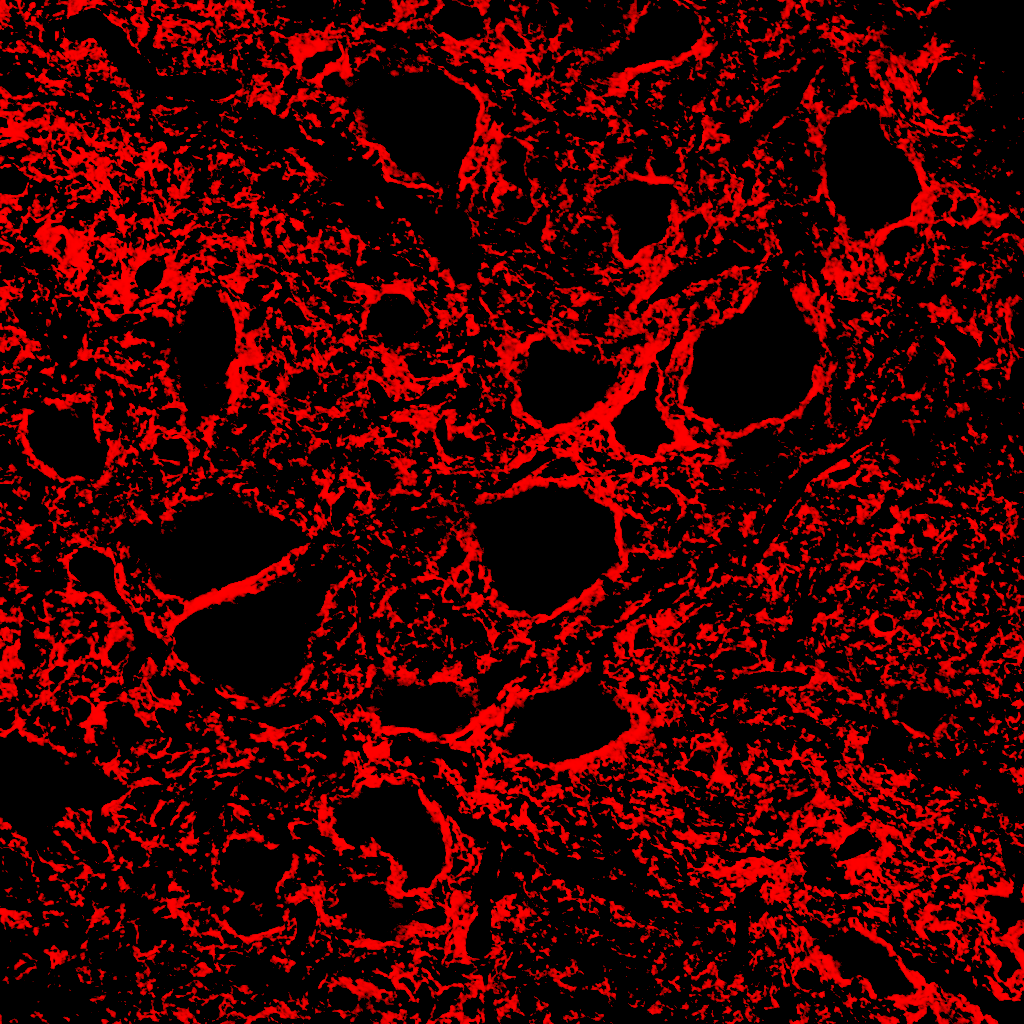

Supplement: Supplementary file 1 [file Data_Sheet_1.zip › Supplementary materials/Immunofluorescence images/Group C/AQP4.tif]

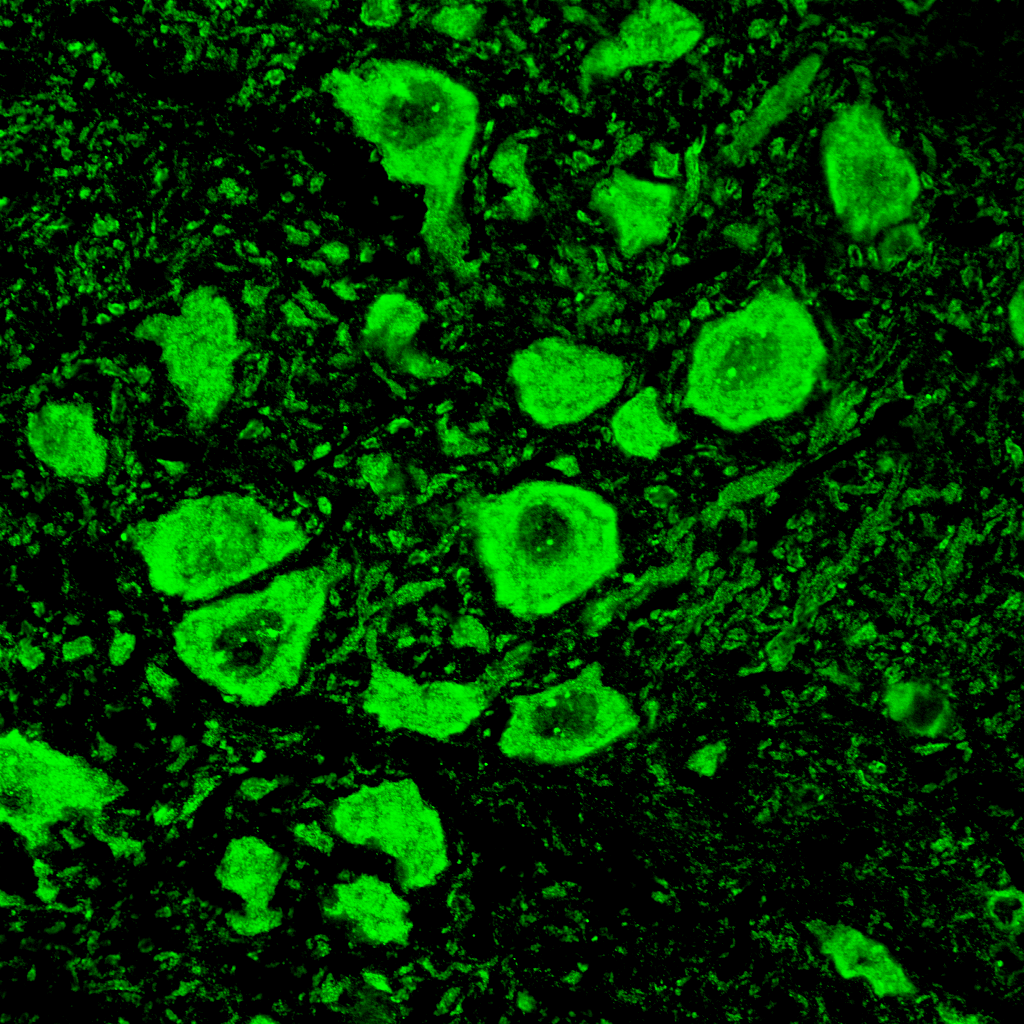

Supplement: Supplementary file 1 [file Data_Sheet_1.zip › Supplementary materials/Immunofluorescence images/Group C/CD31.tif]

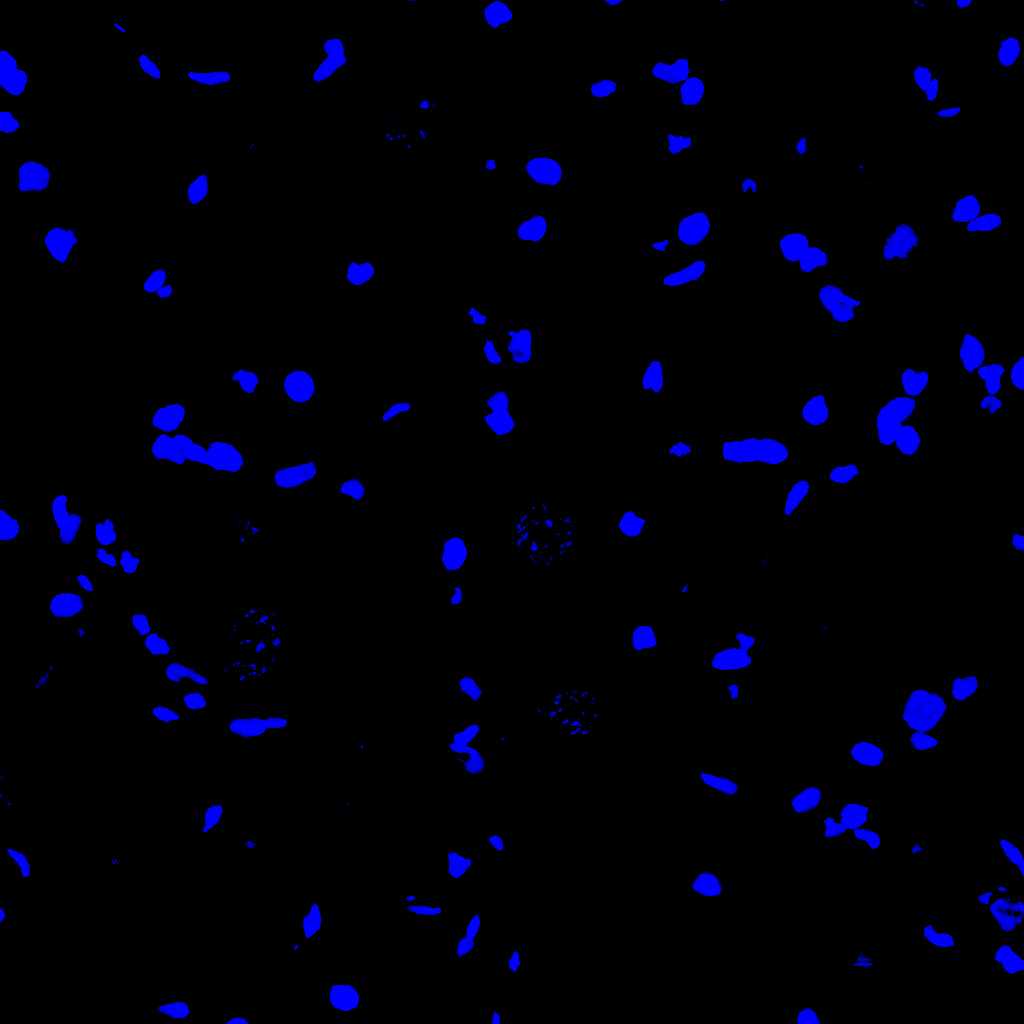

Supplement: Supplementary file 1 [file Data_Sheet_1.zip › Supplementary materials/Immunofluorescence images/Group C/DAPI.tif]

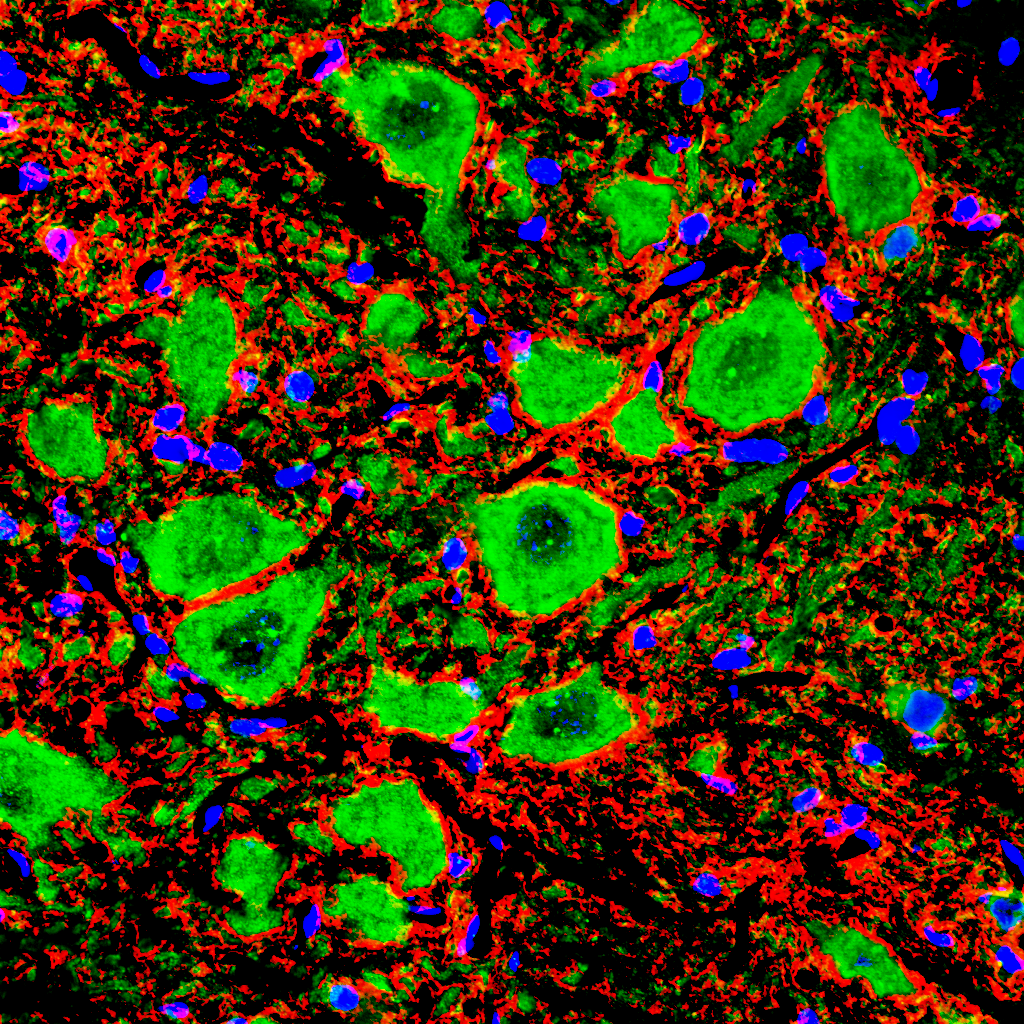

Supplement: Supplementary file 1 [file Data_Sheet_1.zip › Supplementary materials/Immunofluorescence images/Group C/Merge.tif]

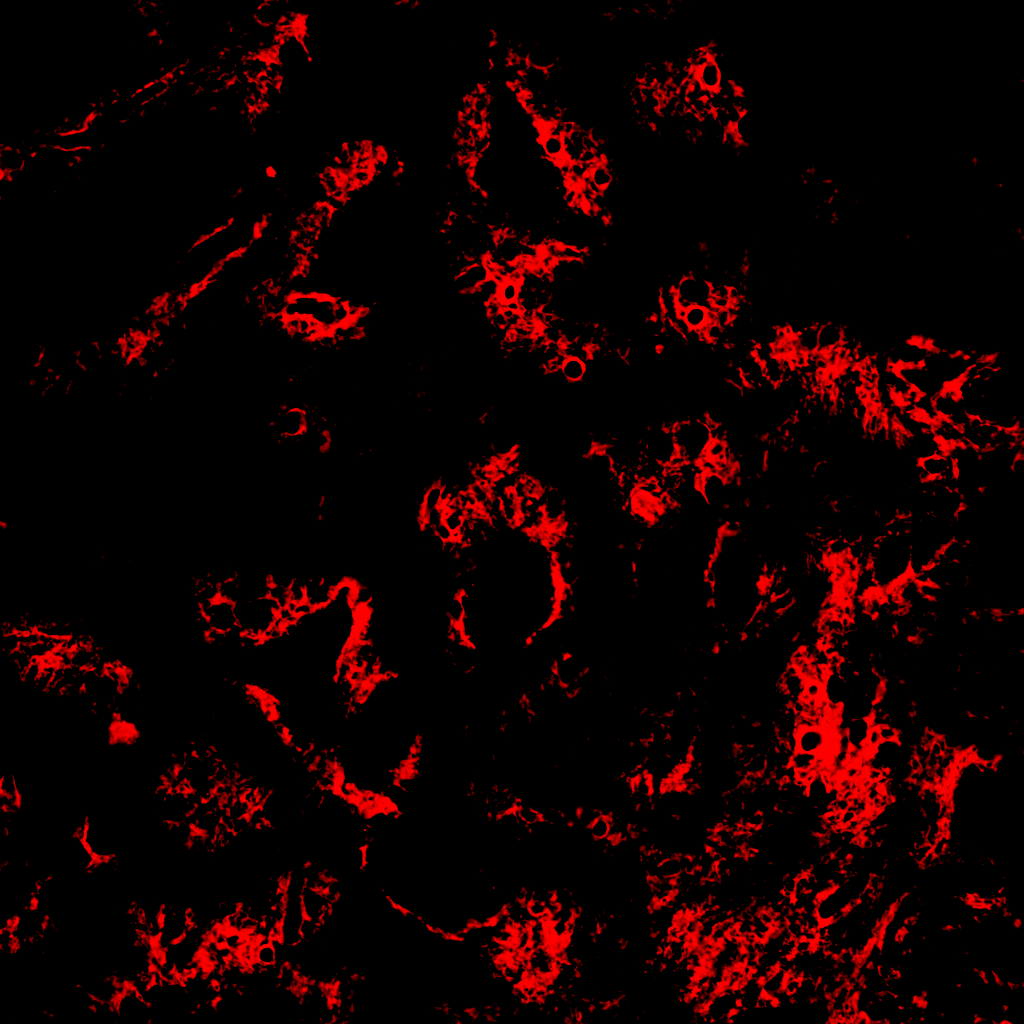

Supplement: Supplementary file 1 [file Data_Sheet_1.zip › Supplementary materials/Immunofluorescence images/Group PDN/AQP4.tif]

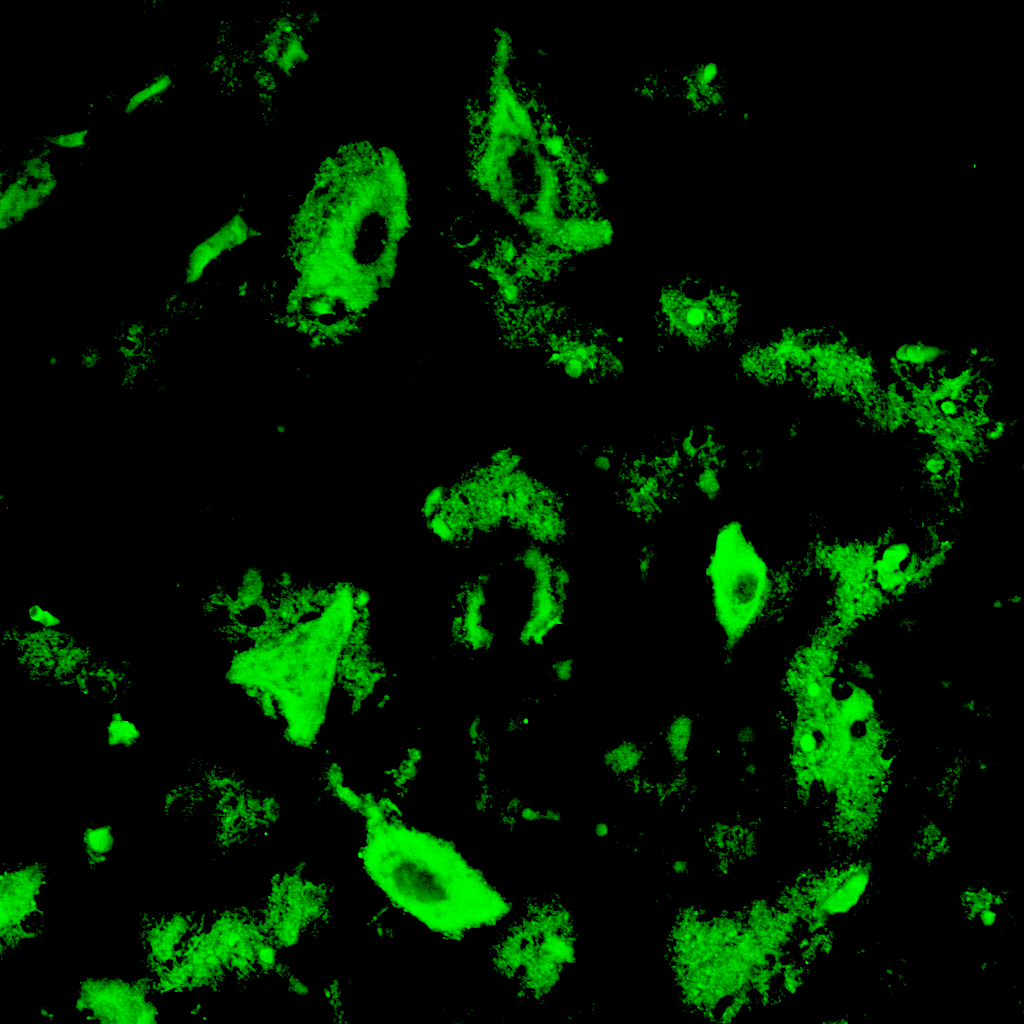

Supplement: Supplementary file 1 [file Data_Sheet_1.zip › Supplementary materials/Immunofluorescence images/Group PDN/CD31.tif]

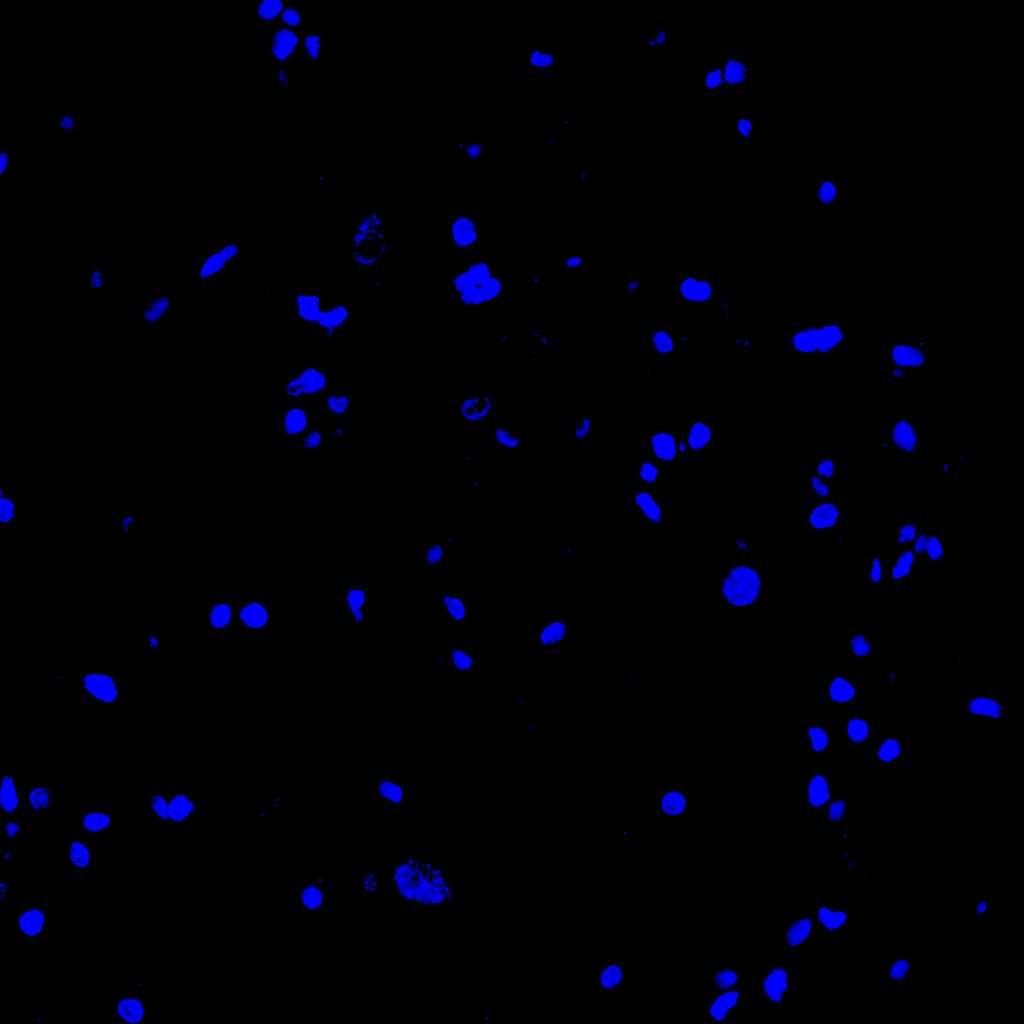

Supplement: Supplementary file 1 [file Data_Sheet_1.zip › Supplementary materials/Immunofluorescence images/Group PDN/DAPI.tif]

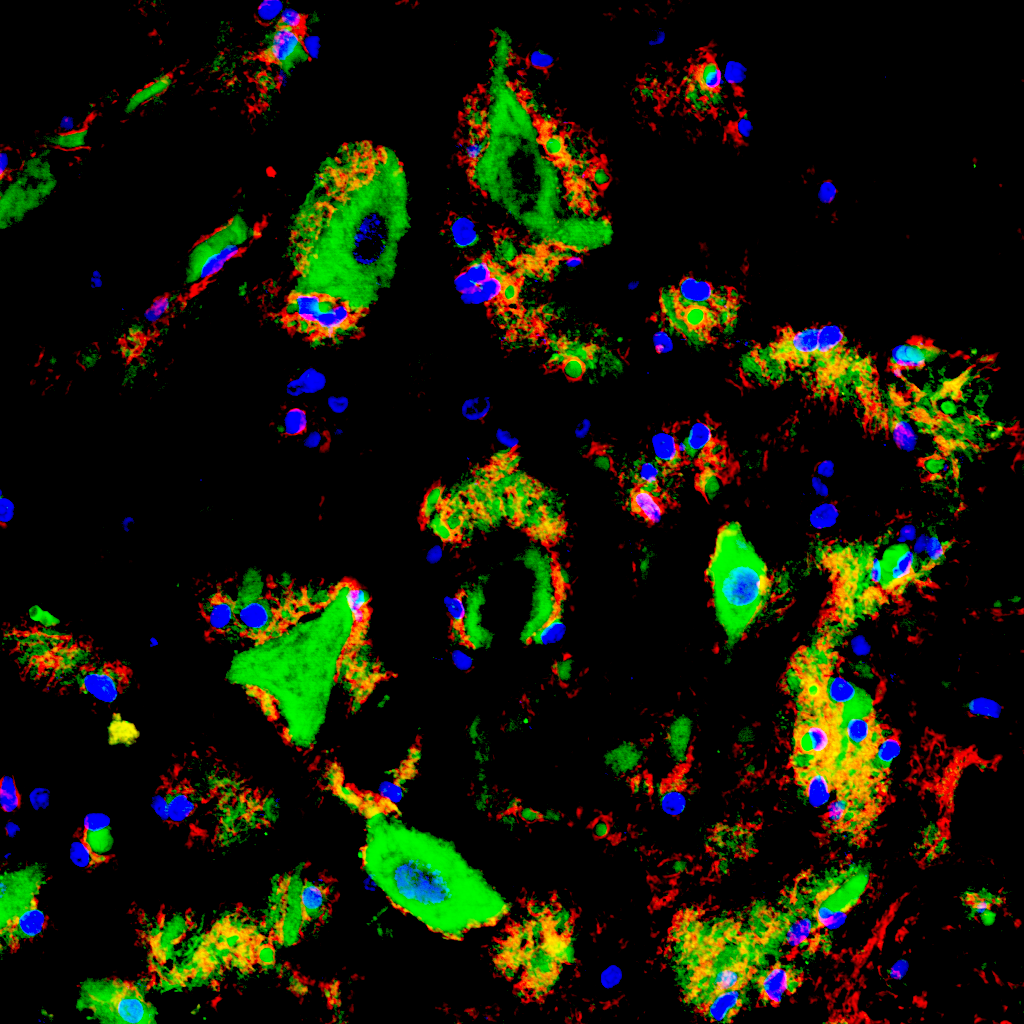

Supplement: Supplementary file 1 [file Data_Sheet_1.zip › Supplementary materials/Immunofluorescence images/Group PDN/Merge.tif]

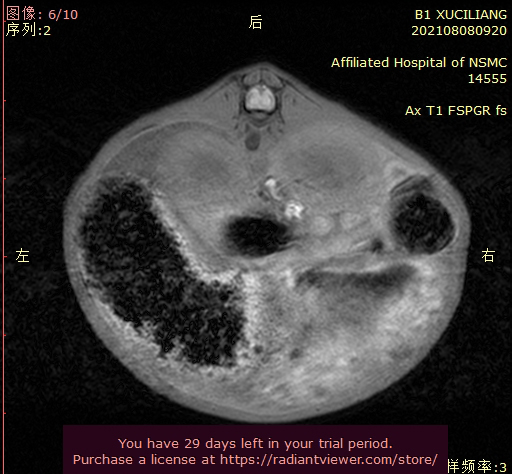

Supplement: Supplementary file 1 [file Data_Sheet_1.zip › Supplementary materials/MRI images/Group BHB/BHB 0.5h.jpg]

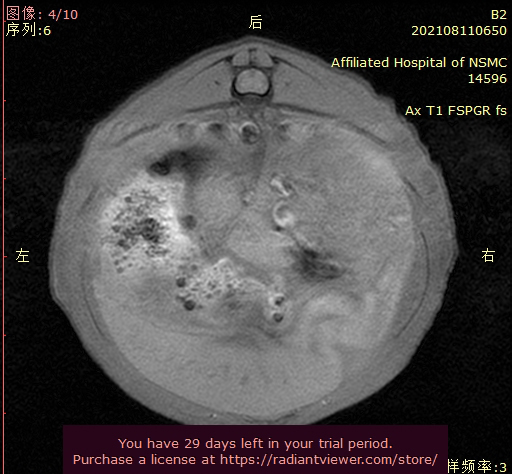

Supplement: Supplementary file 1 [file Data_Sheet_1.zip › Supplementary materials/MRI images/Group BHB/BHB 0min.jpg]

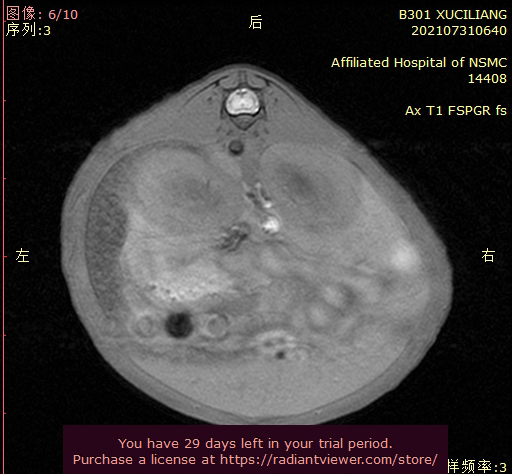

Supplement: Supplementary file 1 [file Data_Sheet_1.zip › Supplementary materials/MRI images/Group BHB/BHB 1.5h.jpg]

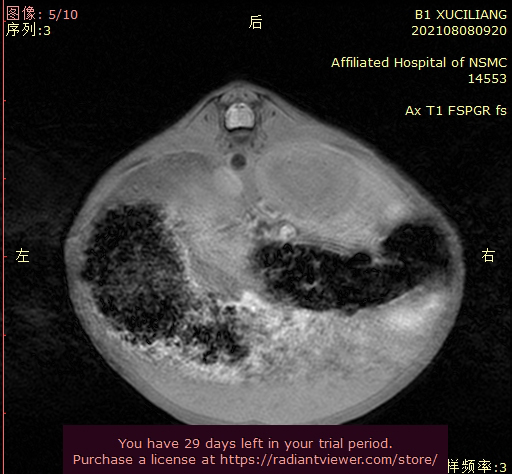

Supplement: Supplementary file 1 [file Data_Sheet_1.zip › Supplementary materials/MRI images/Group BHB/BHB 15min.jpg]

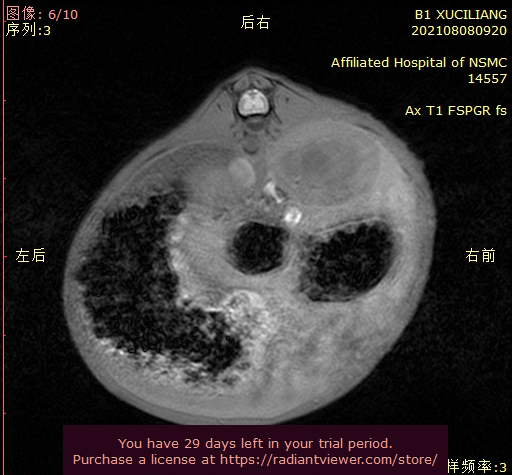

Supplement: Supplementary file 1 [file Data_Sheet_1.zip › Supplementary materials/MRI images/Group BHB/BHB 1h.jpg]

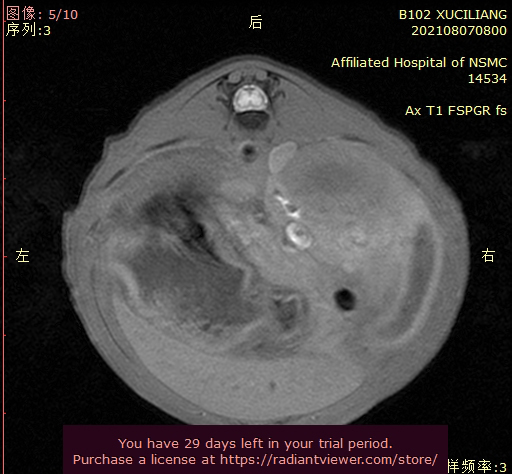

Supplement: Supplementary file 1 [file Data_Sheet_1.zip › Supplementary materials/MRI images/Group BHB/BHB 2.5h.jpg]

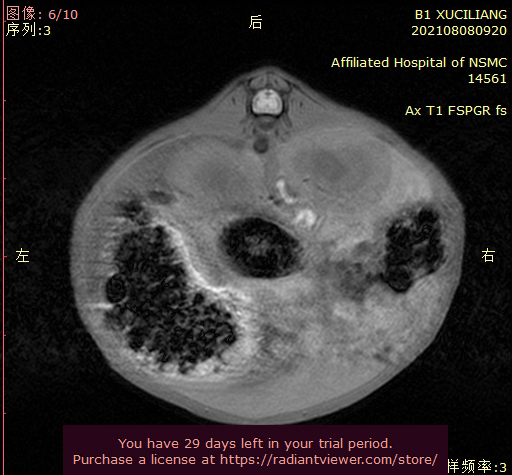

Supplement: Supplementary file 1 [file Data_Sheet_1.zip › Supplementary materials/MRI images/Group BHB/BHB 2h.jpg]

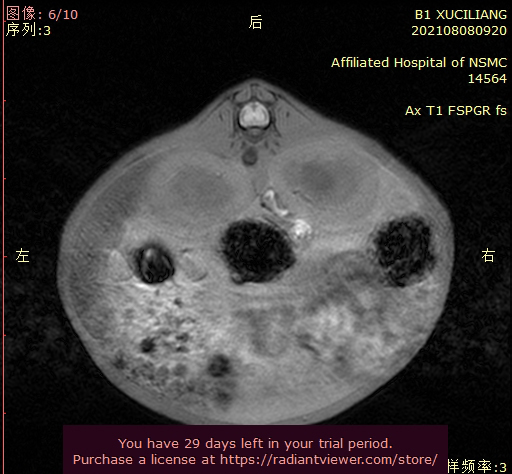

Supplement: Supplementary file 1 [file Data_Sheet_1.zip › Supplementary materials/MRI images/Group BHB/BHB 3h.jpg]

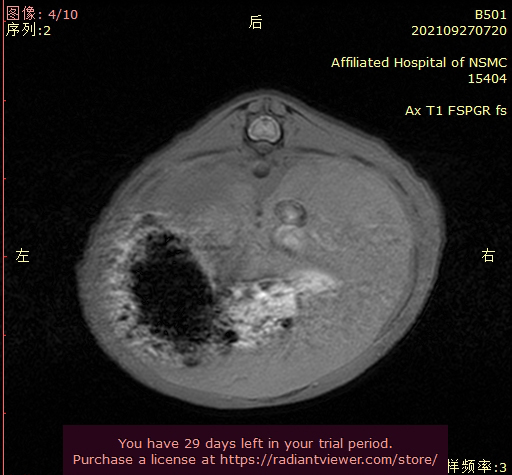

Supplement: Supplementary file 1 [file Data_Sheet_1.zip › Supplementary materials/MRI images/Group BHB/BHB 6h.jpg]

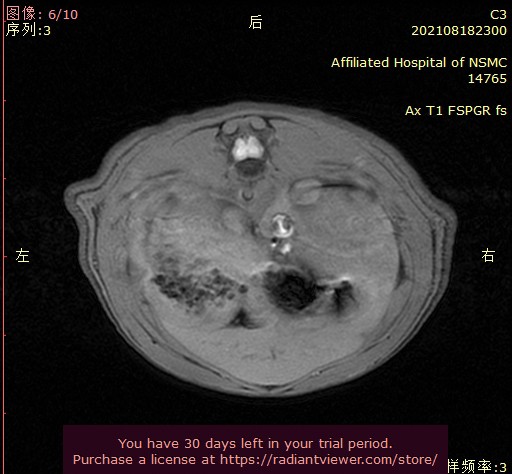

Supplement: Supplementary file 1 [file Data_Sheet_1.zip › Supplementary materials/MRI images/Group C/0.5h.jpg]

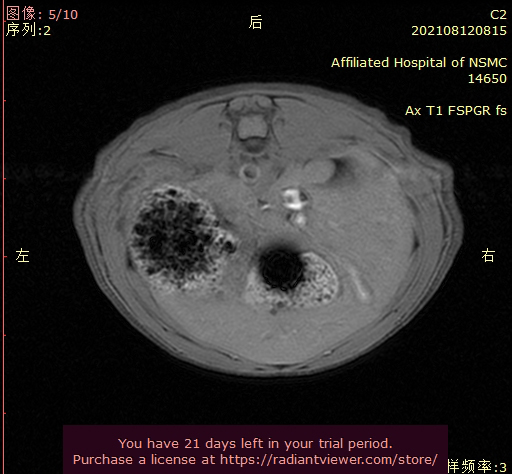

Supplement: Supplementary file 1 [file Data_Sheet_1.zip › Supplementary materials/MRI images/Group C/0min.jpg]

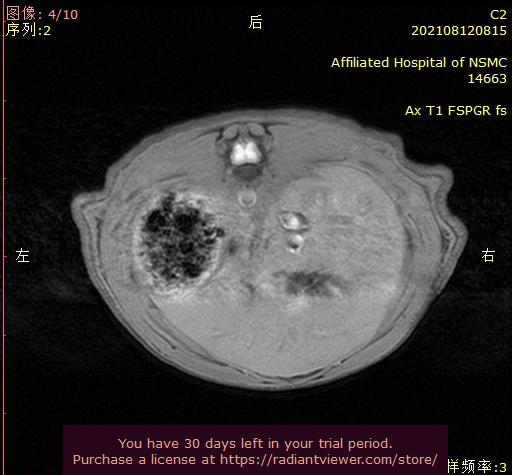

Supplement: Supplementary file 1 [file Data_Sheet_1.zip › Supplementary materials/MRI images/Group C/1.5h.jpg]

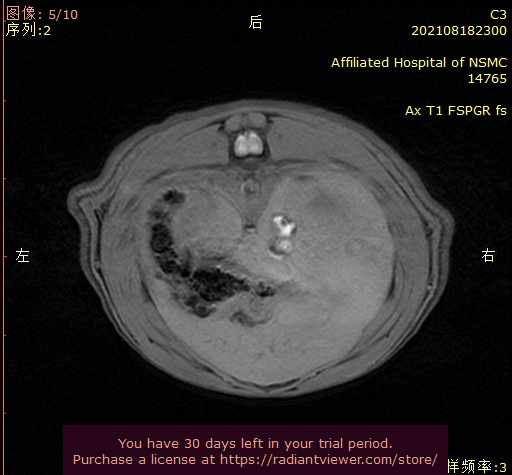

Supplement: Supplementary file 1 [file Data_Sheet_1.zip › Supplementary materials/MRI images/Group C/15min.jpg]

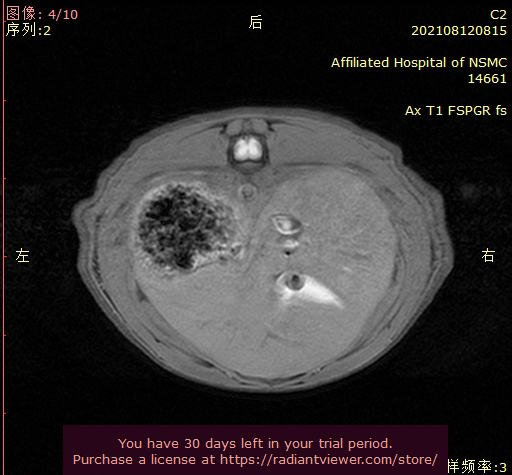

Supplement: Supplementary file 1 [file Data_Sheet_1.zip › Supplementary materials/MRI images/Group C/1h.jpg]

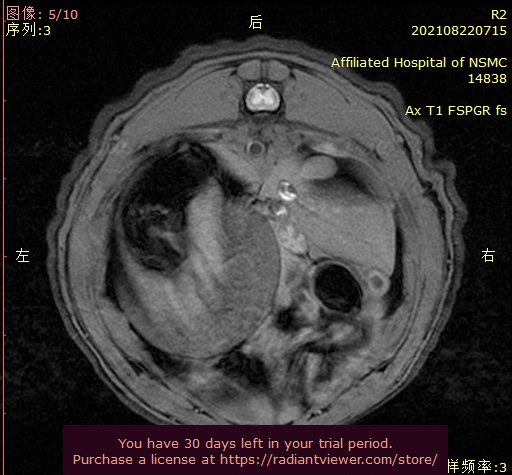

Supplement: Supplementary file 1 [file Data_Sheet_1.zip › Supplementary materials/MRI images/Group C/2.5h.jpg]

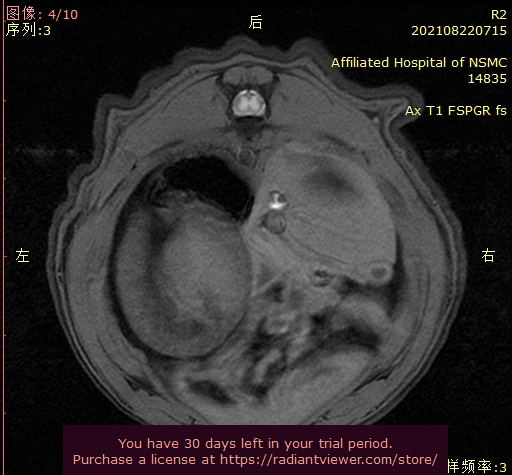

Supplement: Supplementary file 1 [file Data_Sheet_1.zip › Supplementary materials/MRI images/Group C/2h.jpg]

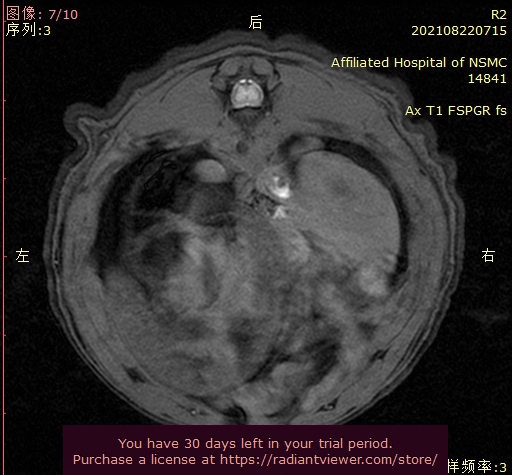

Supplement: Supplementary file 1 [file Data_Sheet_1.zip › Supplementary materials/MRI images/Group C/3h.jpg]

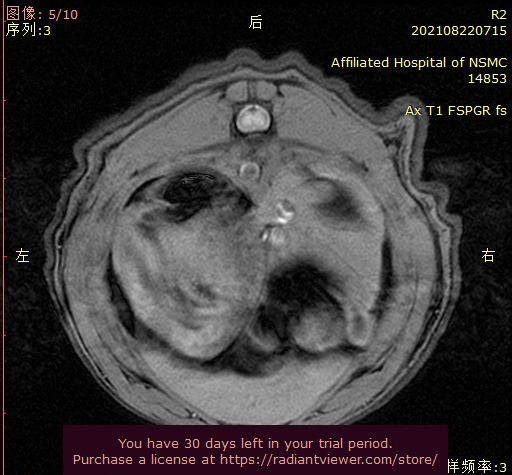

Supplement: Supplementary file 1 [file Data_Sheet_1.zip › Supplementary materials/MRI images/Group C/6h.jpg]

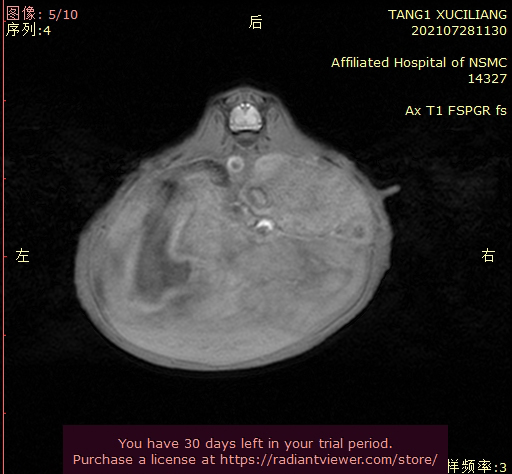

Supplement: Supplementary file 1 [file Data_Sheet_1.zip › Supplementary materials/MRI images/Group PDN/PDN 0.5h.jpg]

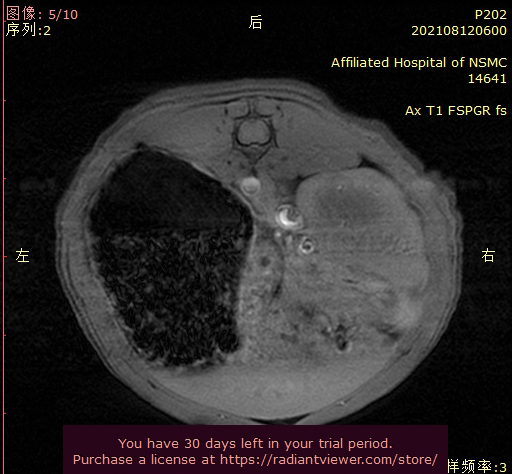

Supplement: Supplementary file 1 [file Data_Sheet_1.zip › Supplementary materials/MRI images/Group PDN/PDN 0min.jpg]

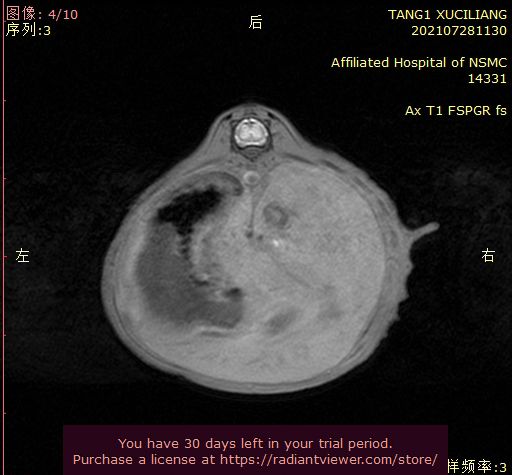

Supplement: Supplementary file 1 [file Data_Sheet_1.zip › Supplementary materials/MRI images/Group PDN/PDN 1.5h.jpg]

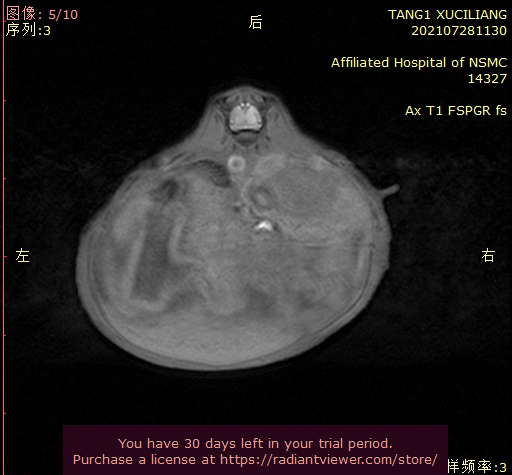

Supplement: Supplementary file 1 [file Data_Sheet_1.zip › Supplementary materials/MRI images/Group PDN/PDN 15min.jpg]

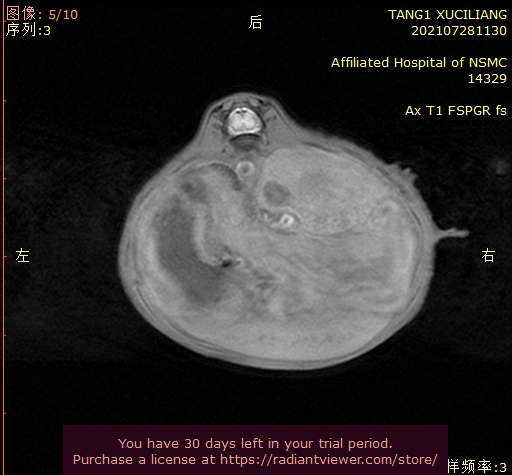

Supplement: Supplementary file 1 [file Data_Sheet_1.zip › Supplementary materials/MRI images/Group PDN/PDN 1h.jpg]

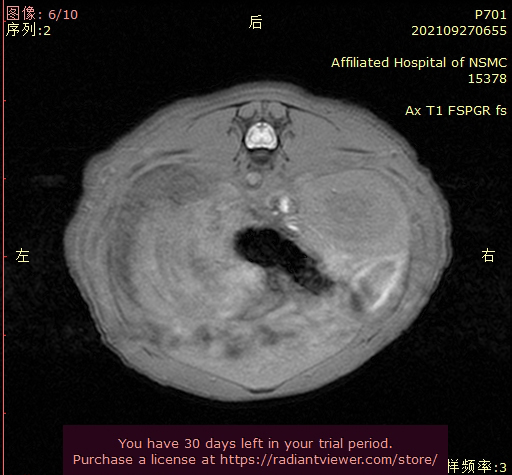

Supplement: Supplementary file 1 [file Data_Sheet_1.zip › Supplementary materials/MRI images/Group PDN/PDN 2.5h.jpg]

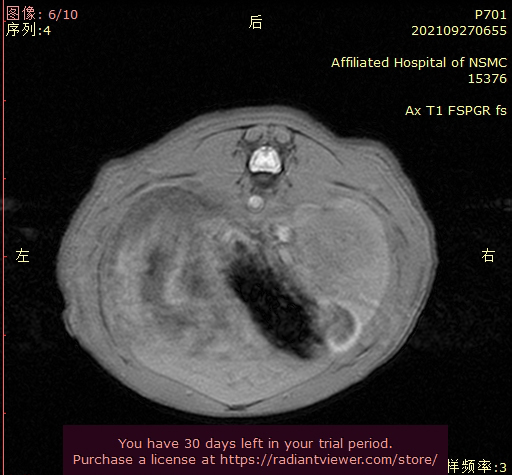

Supplement: Supplementary file 1 [file Data_Sheet_1.zip › Supplementary materials/MRI images/Group PDN/PDN 2h.jpg]

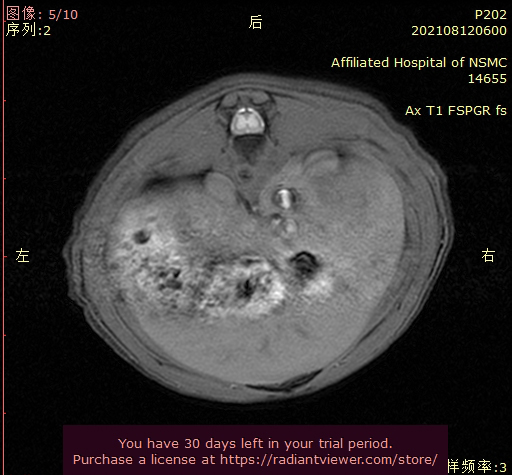

Supplement: Supplementary file 1 [file Data_Sheet_1.zip › Supplementary materials/MRI images/Group PDN/PDN 3h.jpg]

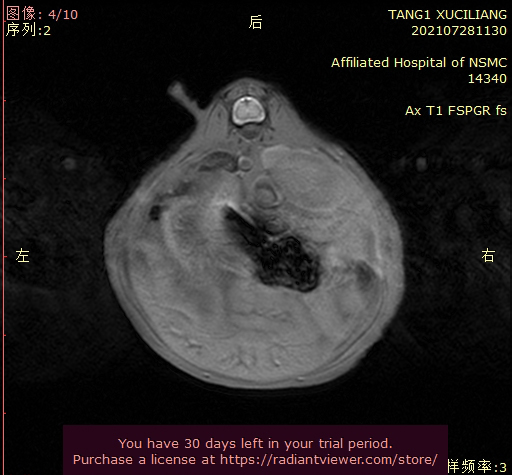

Supplement: Supplementary file 1 [file Data_Sheet_1.zip › Supplementary materials/MRI images/Group PDN/PDN 6h.jpg]

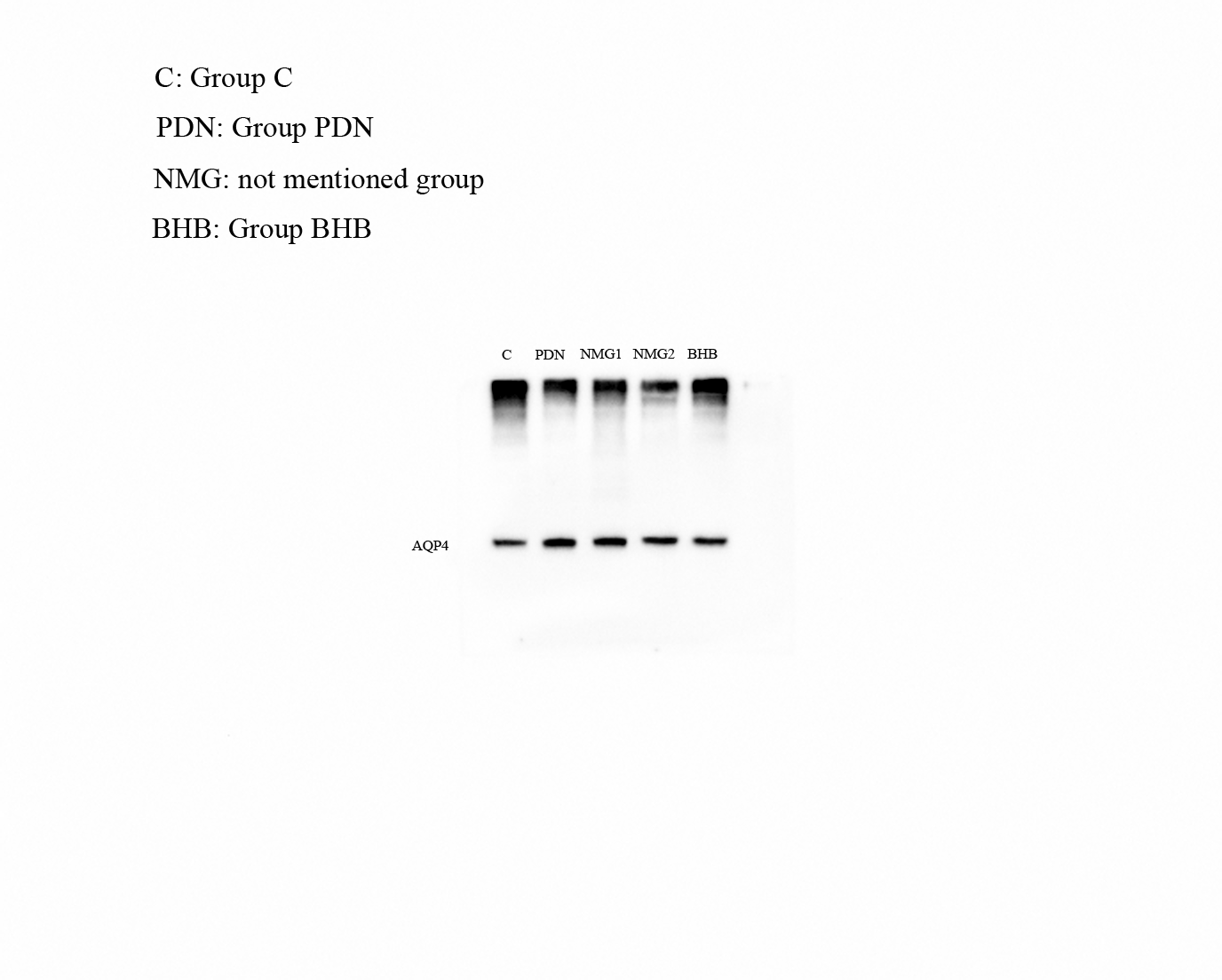

Supplement: Supplementary file 1 [file Data_Sheet_1.zip › Supplementary materials/WB original results/AQP4.Tif]

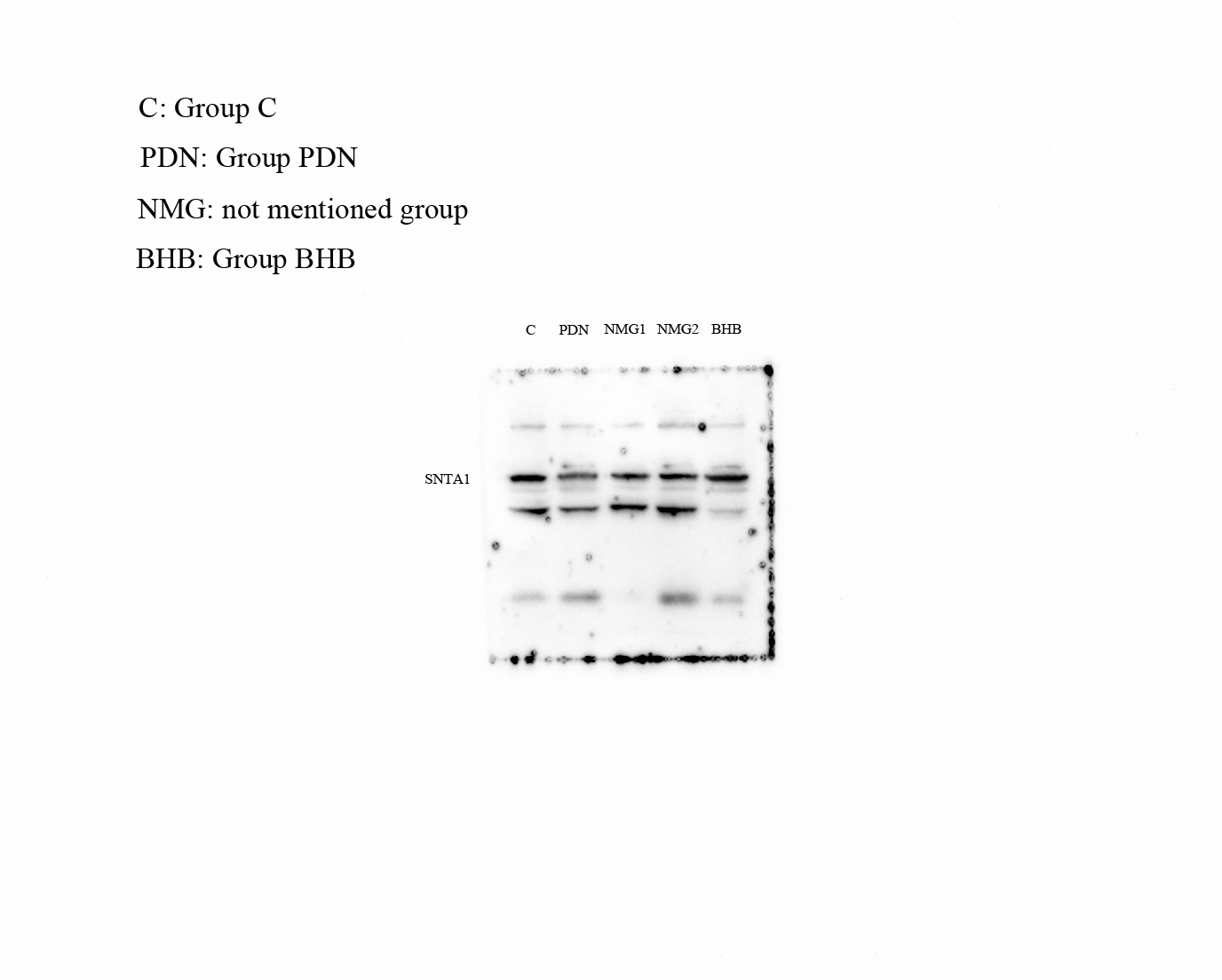

Supplement: Supplementary file 1 [file Data_Sheet_1.zip › Supplementary materials/WB original results/SNTA1.Tif]

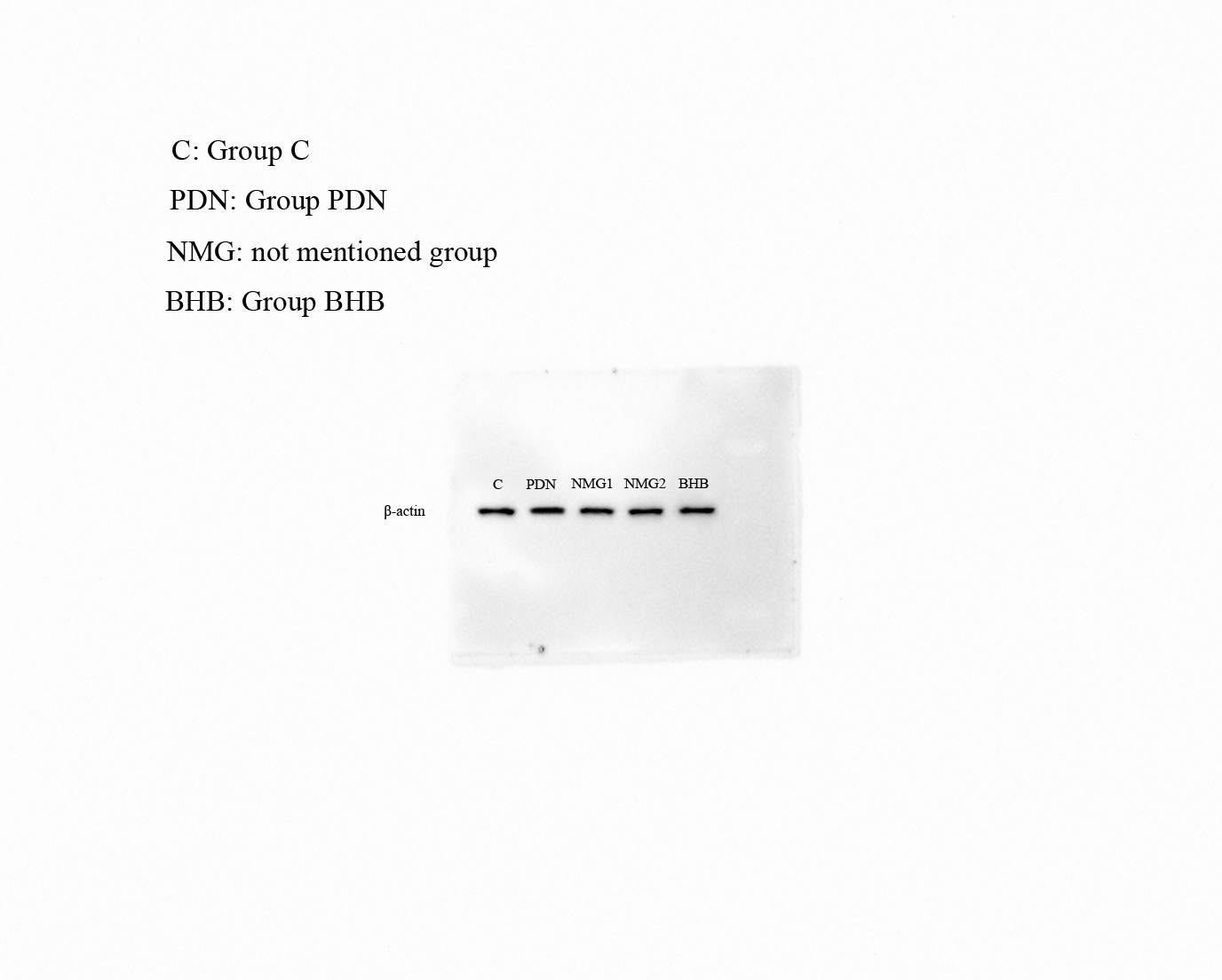

Supplement: Supplementary file 1 [file Data_Sheet_1.zip › Supplementary materials/WB original results/β-actin.Tif]
