# Supplementary material for: β-Hydroxybutyrate Attenuates Painful Diabetic Neuropathy via Restoration of the Aquaporin-4 Polarity in the Spinal Glymphatic System
Source: Front Neurosci. 2022 Jul 11;16:926128. doi: 10.3389/fnins.2022.926128 (PMC9309893; doi:10.3389/fnins.2022.926128)
Supplement: Supplementary file 1 [file Data_Sheet_1.zip › Supplementary materials/WB original results/Statement.doc]

Dear Editor

Thanks for your concerns about our manuscript. This is a statement of why we spliced the western blot images. Due to the reason that some blots in the original saved images were not mentioned in the present study, we spliced the blots and the typical ones were chosen to indicate the differences in the expression of proteins.

The blots not mentioned in the present study were two groups of rats. NMG1 is a group of rats with painful diabetic neuropathy, and they sacrificed at the time when their paw withdrawal time were at the beginning of the decrease. NMG2 is a group of rats with increased blood glucose but with no decrease in paw withdrawal threshold. We mainly try to explore whether there might be differences between these rats.

In consideration that the animal model at that time may be in an unstable state and was not the main objective rats, and we only did the MRI experiment once, so we only mentioned the results of the western blot in rats with a stable state and concordant with the MRI experiment, in the present study.

we highly appreciate your time and consideration.

Sincerely.

Jing-yan Lin
